# Anticancer Effects of New Ceramides Isolated from the Red Sea Red Algae *Hypnea* *musciformis* in a Model of Ehrlich Ascites Carcinoma: LC-HRMS Analysis Profile and Molecular Modeling

**DOI:** 10.3390/md20010063

**Published:** 2022-01-10

**Authors:** Sameh S. Elhady, Eman S. Habib, Reda F. A. Abdelhameed, Marwa S. Goda, Reem M. Hazem, Eman T. Mehanna, Mohamed A. Helal, Khaled M. Hosny, Reem M. Diri, Hashim A. Hassanean, Amany K. Ibrahim, Enas E. Eltamany, Usama Ramadan Abdelmohsen, Safwat A. Ahmed

**Affiliations:** 1 Department of Natural Products, Faculty of Pharmacy, King Abdulaziz University, Jeddah 21589, Saudi Arabia; ssahmed@kau.edu.sa; 2 Department of Pharmacognosy, Faculty of Pharmacy, Suez Canal University, Ismailia 41522, Egypt; emy_197@hotmail.com (E.S.H.); marwa_saeed@pharm.suez.edu.eg (M.S.G.); hashem_omar@pharm.suez.edu.eg (H.A.H.); amany_mohamed@pharm.suez.edu.eg (A.K.I.); enastamany@gmail.com (E.E.E.); 3 Department of Pharmacognosy, Faculty of Pharmacy, Galala University, New Galala 43713, Egypt; reda.fouad@gu.edu.eg; 4 Department of Pharmacology and Toxicology, Faculty of Pharmacy, Suez Canal University, Ismailia 41522, Egypt; reem_ahmed@pharm.suez.edu.eg; 5 Department of Biochemistry, Faculty of Pharmacy, Suez Canal University, Ismailia 41522, Egypt; eman.taha@pharm.suez.edu.eg; 6 Biomedical Sciences Program, University of Science and Technology, Zewail City of Science and Technology, October Gardens, 6th of October, Giza 12578, Egypt; mohamed_hilal@pharm.suez.edu.eg; 7 Department of Medicinal Chemistry, Faculty of Pharmacy, Suez Canal University, Ismailia 41522, Egypt; 8 Department of Pharmaceutics, Faculty of Pharmacy, King Abdulaziz University, Jeddah 21589, Saudi Arabia; kmhomar@kau.edu.sa; 9 Department of Pharmacy Practice, Faculty of Pharmacy, King Abdulaziz University, Jeddah 21589, Saudi Arabia; rdiri@kau.edu.sa; 10 Department of Pharmacognosy, Faculty of Pharmacy, Minia University, Minia 61519, Egypt; usama.ramadan@mu.edu.eg; 11 Department of Pharmacognosy, Faculty of Pharmacy, Deraya University, New Minia 61111, Egypt

**Keywords:** ceramides, MCF-7, Ehrlich ascites carcinoma, VEGF-B, TNF-α, midkine, apoptosis, p53, LC-ESI-HRMS, *Hypnea* *musciformis*

## Abstract

Different classes of phytochemicals were previously isolated from the Red Sea algae *Hypnea* *musciformis* as sterols, ketosteroids, fatty acids, and terpenoids. Herein, we report the isolation of three fatty acids—docosanoic acid **4**, hexadecenoic acid **5**, and alpha hydroxy octadecanoic acid **6**—as well as three ceramides—**A** (**1**), **B** (**2**), and **C** (**3**)—with 9-methyl-sphinga-4,8-dienes and phytosphingosine bases. Additionally, different phytochemicals were determined using the liquid chromatography coupled with electrospray ionization high-resolution mass spectrometry (LC-ESI-HRMS) technique. Ceramides **A** (**1**) and **B** (**2**) exhibited promising in vitro cytotoxic activity against the human breast adenocarcinoma (MCF-7) cell line when compared with doxorubicin as a positive control. Further in vivo study and biochemical estimation in a mouse model of Ehrlich ascites carcinoma (EAC) revealed that both ceramides **A** (**1**) and **B** (**2**) at doses of 1 and 2 mg/kg, respectively, significantly decreased the tumor size in mice inoculated with EAC cells. The higher dose (2 mg/kg) of ceramide **B** (**2**) particularly expressed the most pronounced decrease in serum levels of vascular endothelial growth factor -B (VEGF-B) and tumor necrosis factor-α (TNF-α) markers, as well as the expression levels of the growth factor midkine in tumor tissue relative to the EAC control group. The highest expression of apoptotic factors, p53, Bax, and caspase 3 was observed in the same group that received 2 mg/kg of ceramide **B** (**2**). Molecular docking simulations suggested that ceramides **A** (**1**) and **B** (**2**) could bind in the deep grove between the H2 helix and the Ser240-P250 loop of p53, preventing its interaction with MDM2 and leading to its accumulation. In conclusion, this study reports the cytotoxic, apoptotic, and antiangiogenic effects of ceramides isolated from the Red Sea algae *Hypnea* *musciformis* in an experimental model of EAC.

## 1. Introduction

Of the three classifications of macroalgae, the chemistry of red algae is more diverse than that of green or brown algae. Therefore, red algae are considered the most important source of many biologically active metabolites in comparison with other algal classes [1,2]. The genus *Hypnea* is one of the widest spread red algae, with economic importance as a source of carrageenan [3]. The *Hypnea* species were extensively assessed for their biological activities. Methanolic extract of *Hypnea flagelliformis* was subjected to the 1,1-diphenyl-2-picrylhydrazyl (DPPH) free radical scavenging assay, and it showed a stronger antioxidant activity compared with the standard quercetin [4]. Likewise, the ethyl acetate fraction showed a significantly higher total phenolic content, DPPH·scavenging activity, H_2_O_2_ scavenging activity, and lipid peroxidation inhibition than its crude extract, fractions of *n*-hexane and dichloromethane, and other methanolic fractions of *Hypnea valentiae*. This study introduced the red seaweeds *Hypnea* sp. to be used as food supplements for increasing shelf-life in the food industry and combating carcinogenesis [5]. Methanol extract of *Hypnea valentiae* inhibited acetylcholinesterase (AChE), and this neuroprotective action is considered a first line in the treatment of dementia [6]. Bitencourt and his colleagues observed that a lectin isolated from the red marine alga *Hypnea cervicornis* possessed antinociceptive and anti-inflammatory activity via interaction with the lectin carbohydrate-binding site. Additionally, the lectin did not show visible signs of toxicity at effective doses [7]. The methanolic extract of the algea *Hypnea esperi* from the Suez Canal region showed potent antibacterial activity toward Gram-positive bacteria that correlated to long chain fatty acids of more than 10 carbon atoms in length, which induced lysis of bacterial protoplasts. In addition, *H. esperi* also exhibited anticoagulant activity by delaying the blood clotting to 120 s in comparison with the control blood’s 40-s clotting time [8]. Moreover, several fatty acids such as palmitic, oleic, pentacosanoic, and hexacosenoic acids, as well as sesquiterpene and sterols, were reported in *Hypnea* [9]. The previously isolated compounds from the genus *Hypnea* can be classified into three categories; sterols and ketosteroids, terpenoids, and polymers as polypeptides and polysaccharides [10,11,12,13,14,15]. Although many biological studies were performed, less research work was performed for the isolation of these pure active compounds. Our study was oriented toward finding out other classes of bioactive compounds that attributed to the previously mentioned pharmacological activities of *Hypnea* sp.

The sphingolipid-signaling pathway is a novel anticancer target system. It has been suggested that sphingolipids play fundamental roles in the regulation of cancer pathogenesis and development [16]. Ceramide serves as a central mediator in sphingolipid metabolism and signaling pathways, regulating many essential cellular responses [17]. Many drugs used in the treatment of cancer are themselves ceramide generators. This property contributes in part to their apoptosis-inducing effects [18]. Consequently, targeting the ceramide-signaling pathway by activating ceramide downstream receptors, inhibiting ceramide-metabolizing enzymes, or exogenously increasing the ceramide levels comprise the novel targets for cancer treatment [19]. In the current work, we aimed to assess the potential antitumor and apoptotic activities of two novel ceramides isolated from *Hypnea musciformis*.

## 2. Results and Discussion

### 2.1. Metabolic Analysis Profile

**Figure 1:**
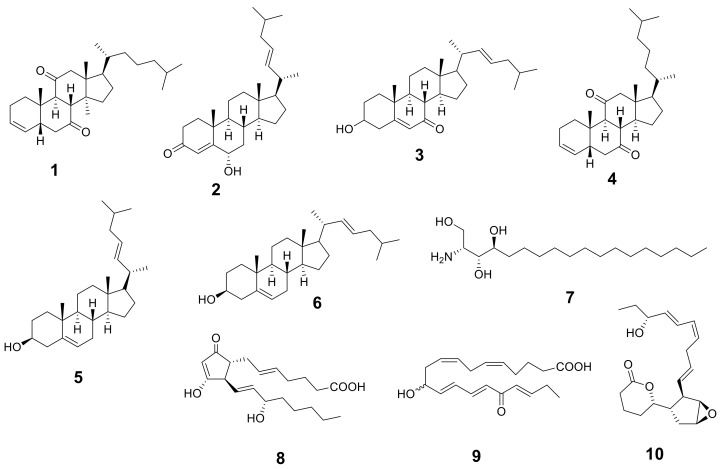
Structures of the identified metabolites listed in Table 1.

The metabolic analysis profile produced by the LC-HR-ESI-MS technique (Figures S1 and S2) manifested different metabolites (Table 1 and Figure 1) that were detected by comparing their detected masses with those recorded in some databases (e.g., the Dictionary of Natural Products (DNP) and Metabolite and Chemical Entity (METLIN)). The mass accuracy was calculated by ((detected mass − expected mass)/expected mass) × 10^6^ and expressed in parts per million (ppm) error [20]. Sterols with cholesterol nucleui were the most common chemical class isolated from *Hypnea musciformis*. Other bioactive metabolites were identified as ptilodene, an antimicrobial and anti-inflammatory icosanoid [21], agardhilactone, an oxylipin with epoxide ring showing anticancer activity [22,23], and oxytocic prostaglandin-E2, which induces labor [24]. Additionally, a phytosphingosine base that exhibited antiphlogistic and antimicrobial activity against Gram-positive and Gram-negative bacteria, viruses, and fungi [25,26] was detected. Therefore, the above-mentioned biological activity may have been related to the identified metabolites.

### 2.2. Identification of Isolated Compounds

**Figure 2:**
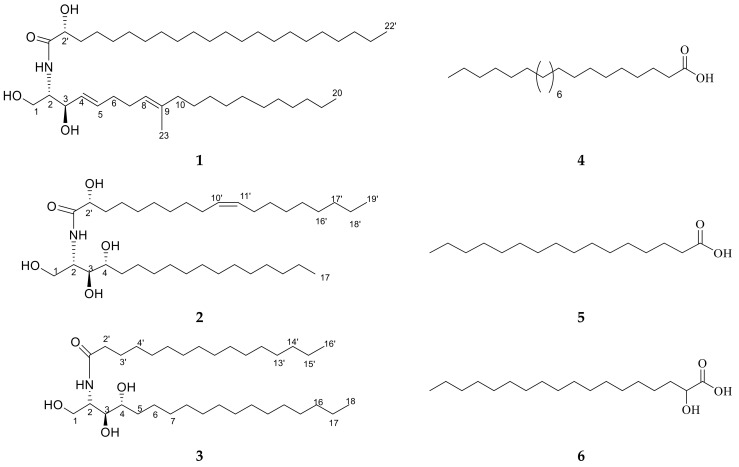
Chemical structures of isolated compounds **1**–**6**.

Ceramide **A** (**1**) (Figure 2) was obtained as a white powder, and its molecular formula was determined to be C_43_H_83_NNaO_4_ by ESI-HRMS with *m*/*z* 700.5921 [M + Na]^+^ (Figure S3), calculated as 700.6220, representing 3 degrees of unsaturation. The ^1^H NMR and ^13^C NMR spectral data of ceramide **A** (**1**) are listed in Table 2 (Figures S4 and S5). The backbone of a ceramide nucleus was recognized by the presence of an amide group at *δ*_H_ 7.26 ppm/*δ*_C_ 175.74 ppm and multiplet peaks of a long methylene chain at *δ*_H_ 1.12–1.32 ppm/*δ*_C_ 29.2–29.7 ppm. An oxygenated methylene was determined at *δ*_H_ 3.75, 4.08 ppm/*δ*_C_ 61.9 ppm. Two oxygenated methine groups as well as a nitrogen-bearing methine were determined at *δ*_H_ 4.08 ppm/*δ*_C_ 74 ppm, *δ*_H_ 4.23 ppm/*δ*_C_ = 72.5 ppm, and *δ*_H_ 3.9 ppm/*δ*_C_ 54.4 ppm, respectively. Four olefinic peaks were detected at *δ*_C_ 129, 134.1, 123.1, and 136.3 ppm and *δ*_H_ 5.51, 5.67, and 5.08 ppm. The length of the fatty acid chain was analyzed by HRMS after performing a protocol of methanolysis [27]. The HRMS showed a molecular ion peak at *m*/*z* 369.3231 [M + H]^+^ (Figure S6), calculated as *m*/*z* 369.3290, indicating a C_22_ fatty acid with a molecular formula of C_23_H_45_O_3_, recognized as 2-hydroxy docosanoic acid methyl ester, while the long chain base was recognized as 1,3-dihydroxy-2-amino-9-methyl-icosene-4,8-diene. Straight chains of both the fatty acid and sphingosine base ended with terminal methyl groups of a normal form at *δ*_H_ 0.88 ppm/*δ*_C_ 14 ppm. Finally, ceramide **A** (**1**) could be identified as a ceramide with alpha-hydroxylated unsaturated fatty acid and long chain of 9-methyl-sphinga-4,8-diene base possessing 2*S*, 2′*R*, 3*R* relative configurations. The configuration of the ceramide moieties was assigned by comparing its physical data, optical rotation ${\left[\alpha \right]}_{D}^{22}$ +6.7 *c* 0.23, CHCl_3_), ^1^H-NMR, and ^13^C-NMR (measured in CDCl_3_) with analogs, using deuterated chloroform as an NMR solvent as reported in the literature [28,29]. The structure of compound **1** was determined to be 2′-hydroxy-N-[(2*S*,2′*R*,3*R*,4*E*,8*E*)-1,3-dihydroxy-9-methyl-icosene-4,8-diene-2-yl]-docosanamide which, to the best of our knowledge, is a new compound.

Ceramide **B** (**2**) (Figure 2) was obtained as a white powder, and its molecular formula was determined to be C_36_H_72_NO_5_ by ESI-HRMS with *m*/*z* 584.4410 [M + H]^+^ (Figure S7), calculated as 584.5176, representing two degrees of unsaturation. The ^1^H NMR and ^13^C NMR spectral data are listed in Table 2 (Figures S8 and S9). The backbone structure of compound **2** was identified as a ceramide. The core of a ceramide nucleus was confirmed by the presence of an amide group at *δ*_C_ 175.1 ppm/*δ*_H_ 8.58 ppm and an overlapped long methylene chain at *δ*_C_ 29.6 ppm/*δ*_H_ 1.22–1.29 ppm. An oxygenated methylene group as well as a nitrogen-bearing methine group were determined at *δ*_H_ 4.40, 4.5 ppm/*δ*_C_ 61.7 ppm and *δ*_H_ 5.11 ppm/*δ*_C_ 52.6 ppm, respectively. Three groups of oxygenated methine were detected at *δ*_C_ 72.1, 72.7, and 76.4 ppm and *δ*_H_ 4.61, 4.28, and 4.35 ppm. Additionally, two olefinic peaks were determined at *δ*_H_ 5.47 ppm/*δ*_C_ 130.0 ppm. Terminal methyl groups of a normal type were detected at *δ*_C_ 13.9 ppm/*δ*_H_ 0.85 ppm. The length of the fatty acid chain was determined on the basis of the results of its oxidative methanolysis followed by peak detection by HRMS, which showed a molecular ion peak at *m*/*z* 313.2709 [M + H]^+^ (Figure S10), calculated as *m*/*z* 313.2743, indicating a methyl ester of C_18:1_ fatty acid with a molecular formula of C_19_H_37_O_3_. Therefore, the fatty ester methyl ester moiety was recognized as 2-hydroxy-10-nonadecenoic acid methyl ester, which was confirmed by GC-MS analysis (Figure S11). At last, ceramide **B** (**2**) could be identified as a ceramide with 2′-hydroxy monounsaturated fatty acid and a long chain phytosphingosine base possessing 2*S*,2′*R*,3*S*,4*R,*9′*Z* relative configurations. The configuration of the ceramide moieties was assigned by comparing its physical data, optical rotation ${\left[\alpha \right]}_{D}^{22}$ +7.70 (*c* 0.27, pyridine), ^1^H-NMR, and ^13^C-NMR (measured in C_5_D_5_N) with the analogs using deuterated pyridine as an NMR solvent, as reported in the literature [27]. The structure of ceramide **B** (**2**) was determined to be 2′-hydroxy-N-[(2*S*,2′*R,*3*S*,4*R,*9′*Z*)-1,3,4-trihydroxy-nonadecan-2-yl]-10-heptadecenamide which, to the best of our knowledge, is a new compound.

Ceramide **C** (**3**) (Figure 2) was obtained as a white powder, and its molecular formula was determined to be C_34_H_70_NO_4_ by ESI-HRMS. The mass spectrum of compound **3** displayed a molecular ion peak with *m*/*z* 556.5660 [M + H]^+^ (Figure S12), calculated as 556.5305, representing one degree of unsaturation. The ^1^H NMR and ^13^C NMR spectral data of ceramide **C** (**3**) are listed in Table 2 (Figures S13 and S14). The backbone structure of compound **3** was determined to be a ceramide, as explained above. In addition, the length of the fatty acid was analyzed by HRMS after its methanolysis. The HRMS showed a molecular ion peak at *m*/*z* 271.9316 [M + H]^+^ (Figure S15), calculated as *m*/*z* 271.2637, indicating a C_16_ fatty acid with a molecular formula of C_17_H_35_O_3_. Therefore, the fatty acid methyl ester moiety was recognized as a palmitic acid methyl ester. Consequently, ceramide **C** (**3**) can be identified as a ceramide with non-hydroxylated saturated fatty acid and a long chain phytosphingosine base possessing 2*S*,3*S*,4*R* relative configurations. The structure of ceramide **C** (**3**) was determined to be N-[(2*S*,3*R*,4*R*)-1,3,4-trihydroxy-octadecan-2-yl] hexadecanamide. The configuration of the ceramide moieties was assigned by comparing its physical data, optical rotation ${\left[\alpha \right]}_{D}^{22}$ +14.30 (*c* 0.25, pyridine), and ^1^H and ^13^C NMR data with the reported data in the literature.

It was found that it was previously isolated under the name (2*S*,3*S*,4*R*)-2-N-(palmitoyl)-phytosphingosine from *Armillaria mellea* [30]. However, it is worth mentioning that ceramide **3** is the first report of this ceramide in *Hypnea musciformis*. Furthermore, it was denoted as ceramide **C** in our biological study.

Other known compounds **4**–**6** (Figure 2) were identified as docosanoic acid (**4**) (Figure S16), hexadecanoic acid (**5**) (Figure S17), and alpha hydroxy octadecanoic acid (**6**) (Figure S18) by comparing the NMR data with the literature [30].

### 2.3. In Vitro Cytotoxic Activity of Isolated Ceramides **A** (**1**), **B** (**2**), and **C** (**3**)

The anticancer activity of ceramides was previously reported against different malignant cell lines [31,32,33,34,35]. The selection of the human breast adenocarcinoma (MCF-7) cell line was based on the global prevalence of breast cancer as well as common side effects of anticancer drugs that may be relatively ineffective against some phases [36]. From Table 3, it was noticed that ceramides **A** (**1**) and **B** (**2**) showed higher in vitro cytotoxic activity than ceramide **C** (**3**) against the MCF-7 cell line. Both ceramides **A** (**1**) and **B** (**2**) exhibited a promising in vitro cytotoxic activity with IC_50_ of 11.07 ± 0.23 µM and 10.17 ± 0.15 µM, respectively, when compared with doxorubicin as a positive control with IC_50_ of 8.65 ± 0.03 µM. The weak in vitro cytotoxic activity of ceramide **C** could be attributed to the absence of an olefinic group and hydroxy fatty acid, in addition to a fatty acyl chain of a shorter length.

The 2-hydroxy fatty acid, like the 2-hydroxy oleic acid, possessed antitumor activity against several types of cancer. Aside from this, the hydroxylation of fatty acids at C2 made some cancer cells sensitive to the antitumor drug [37]. Therefore, further assessment of the in vivo cytotoxic activity of ceramides **A** (**1**) and **B** (**2**) was performed.

### 2.4. The Antitumor Effects of Isolated Ceramides **A** (**1**) and **B** (**2**) in a Mouse Model of Ehrlich Ascites Carcinoma (EAC)

#### 2.4.1. Effect of the Investigated Ceramides on Liver and Kidney Function Markers

The serum levels of the liver enzymes alanine aminotransferase (ALT) and aspartate aminotransferase (AST), as well as the kidney markers urea and creatinine, were determined in all the study groups to assess the possible toxicity of the investigated ceramides in the liver and kidneys. The results showed slightly higher levels of kidney and liver markers in the EAC control group and all the treated groups in comparison with the normal group but with no significant differences, indicating that the investigated doses of ceramide **A** (**1**) and ceramide **B** (**2**) had no detected toxicity in either the liver or kidneys (Table S1). No other toxic effects were detected in the experimental mice. There were also no observed changes in the behavior of the treated mice nor a marked increase in their mortality rates.

#### 2.4.2. Effect of the Investigated Ceramides on the Tumor Weight

**Figure 3:**
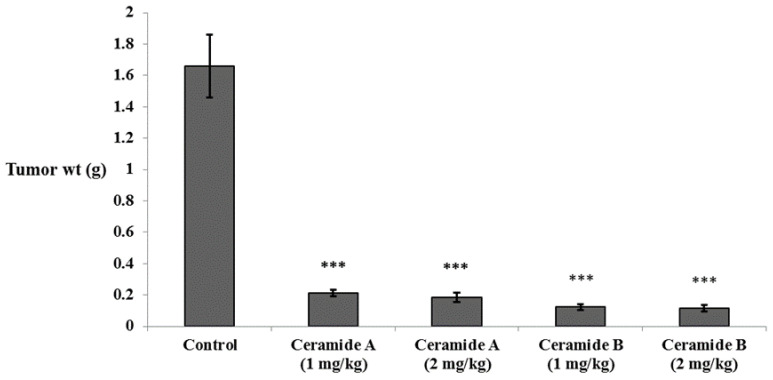
Effect of treatment with ceramide **A** and ceramide **B** at two dose levels (1 mg/kg and 2 mg/kg I.P) on the tumor weight in EAC-bearing mice. Values are expressed as mean ± SD. All data were analyzed using ANOVA followed by a Bonferroni post hoc test. *** Significantly different compared with the EAC control group at *p* < 0.001.

In accordance with the findings of the in vitro study, all groups treated with either ceramide **A** (**1**) or ceramide **B** (**2**) showed a significant decrease in tumor weight compared with the Ehrlich ascites carcinoma (EAC) control group (*p* < 0.001) (Figure 3).

#### 2.4.3. Effect of the Investigated Ceramides on the Serum Levels of Vascular Endothelial Growth Factor B (VEGF-B) and Tumor Necrosis Factor (TNF-α) and the Expression of Midkine (MDK) in the Tumor Tissue

**Figure 4:**
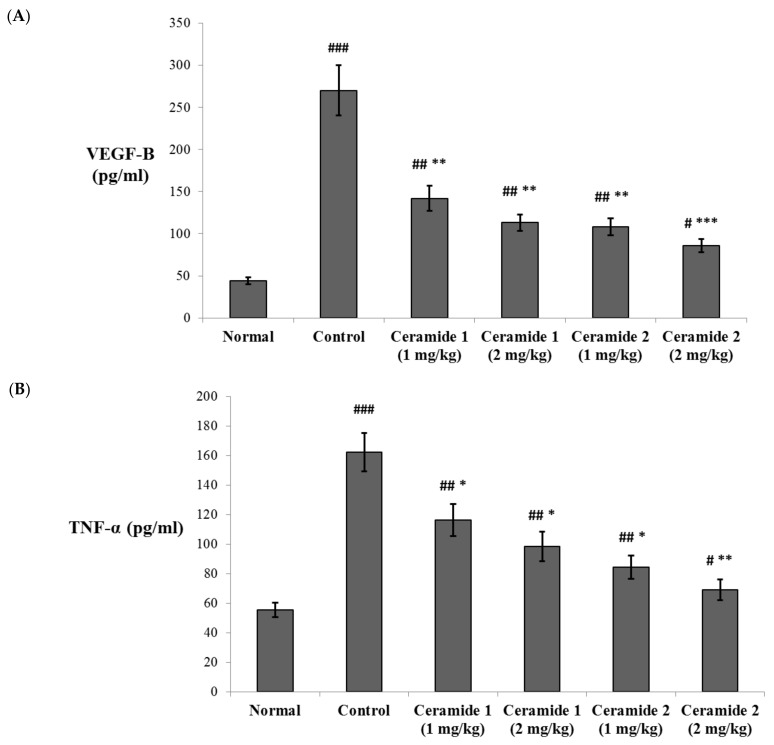
Effect of treatment with ceramide **A** and ceramide **B** at two dose levels (1 mg/kg and 2 mg/kg I.P) on the serum levels of (**A**) vascular endothelial growth factor B (VEGF-B) and (**B**) tumor necrosis factor-α (TNF-α). Values are expressed as mean ± SD. All data were analyzed using ANOVA followed by a Bonferroni post hoc test. ^#^ Significantly different compared with the normal group at *p* < 0.05. ^##^ Significantly different compared with the normal group at *p* < 0.01. ^###^ Significantly different compared with the normal group at *p* < 0.001. * Significantly different compared with the EAC control group at *p* < 0.05. ** Significantly different compared with the EAC control group at *p* < 0.01. *** Significantly different compared with the EAC control group at *p* < 0.001.

The serum levels of vascular endothelial growth factor B (VEGF-B) and the tumor necrosis factor (TNF-α) were assessed by ELISA (Figure 4). Both markers were significantly increased in the EAC control group compared with the normal group (*p* < 0.001). The levels of VEGF-B were significantly decreased upon treatment by ceramide **A** (**1**) (1 and 2 mg/kg) and ceramide **B** (**2**) (1 mg/kg) (*p* < 0.01). The most significant decrease in the levels of VEGF-B in comparison with the EAC control group was recorded in the group treated by 2 mg/kg of ceramide **B** (**2**) (*p* < 0.001) (Figure 4A). Similarly, the levels of TNF-α showed a significant decrease in all treated groups: *p* < 0.05 in groups 3, 4, and 5 (1 and 2 mg/kg of ceramide **A** (**1**) and 1 mg/kg of ceramide **B** (**2**)) and *p* < 0.01 in group 6 (2 mg/kg of ceramide **B** (**2**)) (Figure 4B).

**Figure 5:**
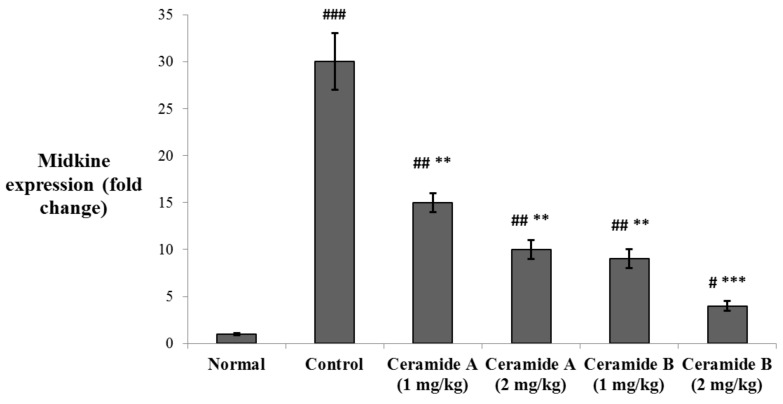
Effect of treatment with ceramide **A** and ceramide **B** at two dose levels (1 mg/kg and 2 mg/kg I.P) on the expression of midkine (MDK) in the tumor tissue. Values are expressed as mean ± SD. All data were analyzed using ANOVA followed by a Bonferroni post hoc test. ^#^ Significantly different compared with the normal group at *p* < 0.05. ^##^ Significantly different compared with the normal group at *p* < 0.01. ^###^ Significantly different compared with the normal group at *p* < 0.001. ** Significantly different compared with the EAC control group at *p* < 0.01. *** Significantly different compared with the EAC control group at *p* < 0.001.

The levels of expression of midkine (MDK) in the tumor tissue were determined by real-time PCR. MDK was significantly upregulated in the EAC control group compared with the normal level (*p* < 0.001). The expression levels were significantly decreased in the groups treated with both doses of ceramide **A** (**1**) (1 and 2 mg/kg) and the lower dose of ceramide **B** (**2**) (1 mg/kg) (*p* < 0.01). The group treated with the higher dose of ceramide **B** (**2**) (2 mg/kg) showed the most significant downregulation of MDK compared with the EAC control group (*p* < 0.001) (Figure 5).

The VEGF members are key promotors of angiogenesis and lymphangiogenesis in malignancies. The level of VEGF-B in plasma was reported as a sensitive marker in breast cancer [38]. Overexpression of VEGF-B was found to promote metastasis in patients with pulmonary [39] and bladder [40] cancers. A higher expression of VEGF-B was also correlated with multiple tumors and positive vascular invasion in hepatocellular carcinoma patients [41]. Zhu et al. [42] suggested that downregulation of VEGF-B signaling may enhance the antitumor effect of resveratrol against pancreatic cancer. VEGF-B acts through binding to vascular endothelial growth factor receptor-1 (VEGFR1), leading to downstream activation of the angiogenetic and proliferative pathways including P38 mitogen-activated protein kinase (p38 MAPK), extracellular signal-regulated kinase (ERK)/MAPK, protein kinase B/serine threonine protein kinase (PKB/AKT), and phosphoinositide 3-kinase (PI3K) [43,44]. On the other hand, the role of TNF-α in cancer has been extensively investigated. It is known to be a double player that has a marked effect in tumor progression on one hand but also may act as a pro-apoptotic agent through activation of the c-Jun N-terminal kinase (JNK) pathway [45,46]. TNF-α is a major pro-inflammatory cytokine secreted by tumor-associated macrophages (TAMs) and by breast cancer cells. It is involved in all stages of the development of breast cancer, including tumor cell proliferation, epithelial-to-mesenchymal transition, metastasis, and recurrence [47]. Higher serum levels of TNF-α were reported in breast cancer patients compared with healthy individuals [48]. Additionally, the levels of TNF-α showed a correlation with the tumor stage in breast cancer patients [49,50,51]. TNF-α also activates nuclear factor kappa B (NFkβ) and induces Jagged1 expression, leading to activation of Notch signaling [52]. In a recent study conducted on a mammary carcinoma model, downregulation of TNF-α was associated with reduced VEGF, interleukin 6 (IL-6), interferon *γ* (IFN-*γ*), Jagged1, and shutting the Notch signaling pathway associated with enhanced apoptosis and declined angiogenesis [53].

MDK is a heparin-binding growth factor that is abnormally expressed in many human cancers, and it was found to mediate several tumor physiological processes involving cell growth, metastasis, migration, and angiogenesis. MDK is considered a key player in cancer progression and is proposed as a potential therapeutic target [54]. MDK expression is induced by cytokines and growth factors, mainly by TNF-α [55], and it acts through activating the NFkβ and MAPK/PI3K proliferative pathway [54]. In clinical studies, MDK was considered a potential prognostic biomarker in solid tumors [56]. In breast cancer patients, it was suggested as both a diagnostic and a prognostic biomarker [57,58]. Interestingly, MDK is a potent proangiogenic factor that promotes tumor angiogenesis [59] and is thought to play a role in controlling the plasma bioavailability of VEGF-A [60]. MDK was also suggested to be implicated in the hypoxia-induced tumor angiogenesis [54,61]. The metastatic effects of MDK are most probably mediated by its combined proinflammatory, angiogenic, and mitogenic functions [62,63,64].

It is noteworthy that the group treated with the higher dose (2 mg/kg) of phytosphingosine ceramide **B** (**2**) expressed the most pronounced decrease in all the biochemically determined markers relative to the EAC control group, as mentioned above. The serum levels of VEGF-B and TNF-α, as well as the expression levels of MDK in the mice treated with 2 mg/kg of ceramide **B** (**2**), revealed the least significant difference compared with the normal (negative control) group (*p* < 0.05). This agrees with the findings of Kwon et al. [65], who reported that a phytosphingosine derivative exhibited an anti-angiogenic effect through markedly decreasing VEGF-induced proteolytic enzyme production, VEGF-induced chemotactic migration, and capillary-like tube formation. 

#### 2.4.4. Effect of the Investigated Ceramides on the Expression of the Apoptotic Markers p35, Bax, and Caspase 3 as Determined by Immunohistochemistry in the Tumor Tissue

**Figure 6:**
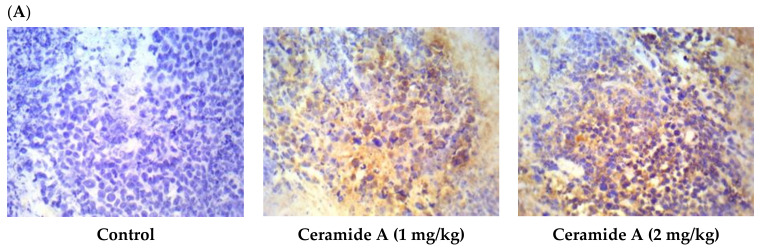

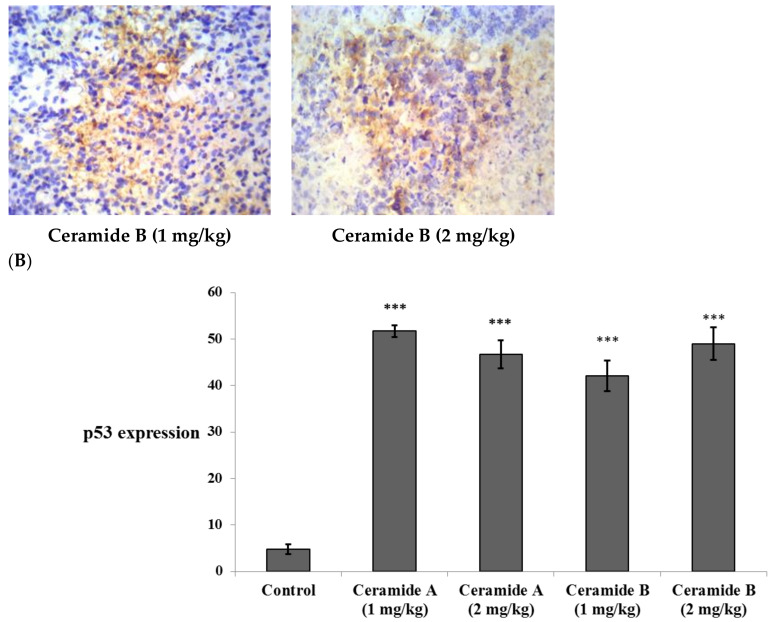
Effect of treatment with ceramide **A** and ceramide **B** at two dose levels (1 mg/kg and 2 mg/kg I.P) on the expression of p53. (**A**) Representative photomicrographs of p53 assessed immunohistochemically on day 21 in EAC-bearing female mice (40× magnification). (**B**) Optical density of positive immunohistochemical reactions (brown), determined using ImageJ. Values are expressed as mean ± SD. All data were analyzed using ANOVA followed by a Bonferroni post hoc test. *** Significantly different compared with the EAC control group at *p* < 0.001.

**Figure 7:**
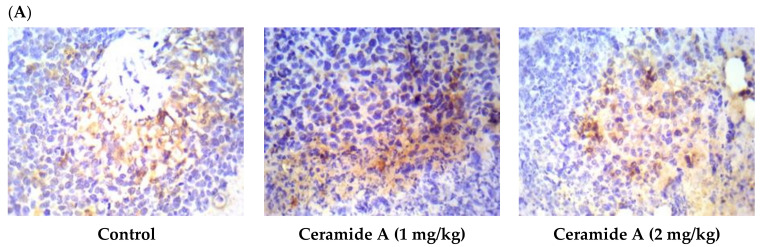

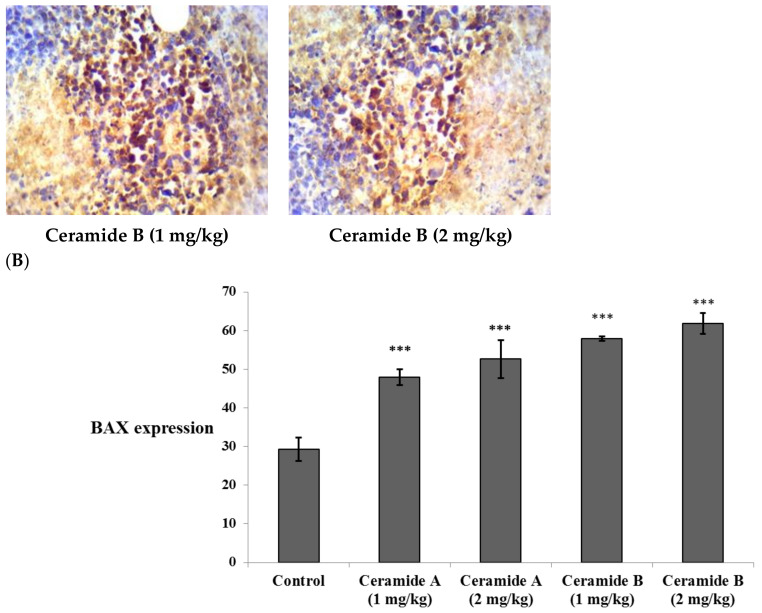
Effect of treatment with ceramide **A** and ceramide **B** at two dose levels (1 mg/kg and 2 mg/kg I.P) on the expression of p53. (**A**) Representative photomicrographs of Bax, assessed immunohistochemically on day 21 in EAC-bearing female mice (40× magnification). (**B**) Optical density of positive immunohistochemical reactions (brown) determined using ImageJ. Values are expressed as mean ± SD. All data were analyzed using ANOVA followed by a Bonferroni post hoc test. *** Significantly different compared with the EAC control group at *p* < 0.001.

Treatment with ceramide **A** (**1**) and **B** (**2**) at both doses augmented the expression of p53, with a significant difference from the control EAC group (*p* < 0.001) (Figure 6). Similarly, the expression of Bax was raised after treatment with both ceramides at the given doses (1 at 2 mg/kg), with a significant difference compared with the control EAC group (*p* < 0.001). The highest expression was observed in the group that received ceramide **B** (**2**) (2 mg/kg) (Figure 7).

The relationship between ceramide and p53 is very complex, and the mechanisms underlying their coregulation are diverse and not fully characterized [66]. The p53 protein was established as a tumor suppressor [67]. It has been assumed that p53 exerts its effect by inducing apoptosis [68]. Cancer research was concerned with both the p53 and ceramide pathways in the regulation of cell growth, cell cycle arrest, and apoptosis [69]. Therefore, the present study investigated the connection between exogenous ceramide uptake and the levels of p53.

An association between p53 and ceramide was observed upon investigation of the cellular response to folate stress. Stressing the A549 cells caused p53-dependent activation of de novo ceramide biosynthesis and C16-ceramide elevation followed by apoptosis [70]. Coadministration of C6-ceramide with vincristine caused apoptosis in multiple cell lines. Significant activation of p53 was detected in these cells, leading to apoptosis [71]. C2-ceramide was shown to induce cell death via elevation of p53, a subsequent increase in the Bax/Bcl-2 ratio, and caspase activation [72].

**Figure 8:**
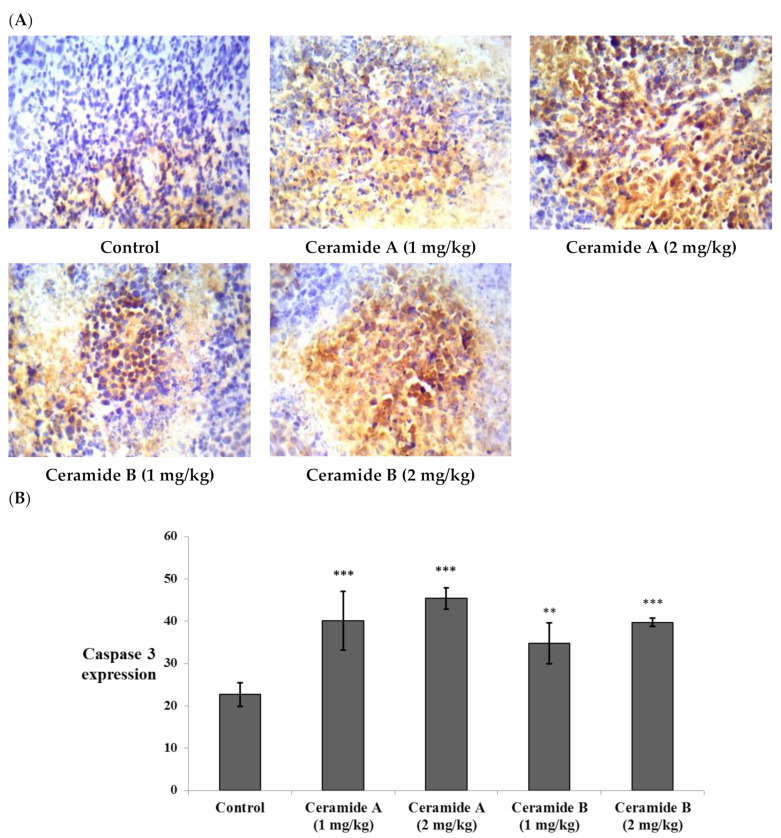
Effect of treatment with ceramide **A** and ceramide **B** at two dose levels (1 mg/kg and 2 mg/kg I.P) on the expression of caspase 3. (**A**) Representative photomicrographs of caspase 3, assessed immunohistochemically on day 21 in EAC-bearing female mice (40× magnification). (**B**) Optical density of positive immunohistochemical reactions (brown) determined using ImageJ. Values are expressed as mean ± SD. All data were analyzed using ANOVA followed by a Bonferroni post hoc test. ** Significantly different compared with the EAC control group at *p* < 0.01. *** Significantly different compared with the EAC control group at *p* < 0.001.

Likewise, the expression of caspase 3 showed significant elevation compared with the control EAC (*p* < 0.001) in all treatment groups (Figure 8). This finding is in agreement with a previous study which reported that phytosphingosine can potently induce apoptotic cell death in human cancer cells via activation of caspase 3, 8, and 9, mitochondrial translocation of Bax, and the subsequent release of cytochrome c into the cytoplasm, providing a potential mechanism for the anticancer activity of phytosphingosine [73]. Additionally, sphingosine was reported to mediate apoptosis in various cancer cell lines through a caspase-dependent mechanism as well as truncation of Bax, which promotes pro-death activity [74].

Ceramide is involved in the induction of apoptosis and growth arrest in breast cancer [75]. The mechanism of ceramide-induced apoptosis involves elevated ceramide levels in the mitochondria, resulting in mitochondrial dysfunction, including a loss of cytochrome c [76]. Mitochondrial apoptosis is dependent on the increased mitochondrial outer membrane permeability. This permeability is enhanced by proteins such as Bax [77]. Therefore, channel formation by ceramide is an upstream event to the induction of apoptosis [78]. Just after the passage of ceramides into the mitochondria, many pro-apoptotic proteins are released into the cytoplasm, primarily cytochrome c [79]. Cytochrome c binds to Apaf-1 (apoptotic protease-activating factor-1). As a result, inactive procaspase-9 is cleaved into active caspase-9. Caspase-9 stimulates caspase-3, which is the crucial step for the caspase cascade in intrinsic apoptosis [80]. The start of intrinsic apoptosis is associated with a rise in mitochondrial ceramide levels [81]. Moreover, exogenous ceramide addition to cells induces apoptosis and DNA fragmentation [82]. Ceramide has been considered a crucial performer in the extrinsic and intrinsic pathways of apoptosis. The extrinsic pathway is activated by the death receptors through interaction with their ligands or by inducing receptor clusterization [83]. Acid sphingomyelinase catalyzes the hydrolysis of sphingomyelin into ceramide, consequently generating ceramide-rich stages and the subsequent clusterization of death receptors that enables the formation of a death-inducing signaling complex and caspase activation [84].

### 2.5. Molecular Modeling

**Figure 9:**
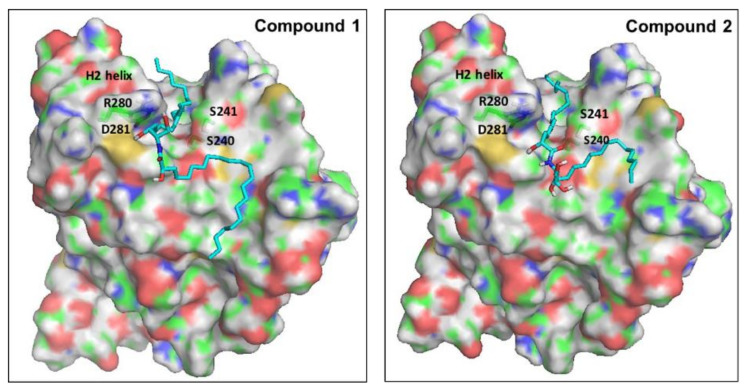
Proposed binding mode of ceramides **1** and **2** into the putative binding site of p53. The surface of the protein is colored by atom type, with carbons in green. Important residues of the binding site are labeled. Docked ligands are shown as cyan sticks.

Ceramides have been considered a group of endogenous sphingolipids with the ability to interfere with the signaling pathways both upstream and downstream of p53 [85]. Under normal non-stressed conditions, the p53 transcription factor undergoes ubiquitination by the E3-ligase mouse double minute 2 (MDM2). This process prepares p53 for the proteasomal degradation. Disruption of the complex between p53 and MDM2 leads to p53 accumulation, nuclear translocation, and activation of the downstream apoptotic pathways [86]. The role of ceramides in the regulation of p53 has remained controversial for a long time, with studies suggesting direct and indirect cross-talk. Recently, in 2018, Fekry et al. from the University of North Carolina showed that C_16_-ceramides could directly interact with p53 [87]. They provided experimental evidence that ceramides bind in close proximity to the BOX V motif of p53, which is a part of the p53–MDM2 interface. Consequently, this binding disrupts the p53-MDM2 binding, reducing the ubiquitination and proteasomal degradation of p53. To gain insights into the molecular determinants of the binding of our ceramides **A** (**1**) and **B** (**2**) into the putative binding site of p53, we decided to perform a molecular docking simulation into the crystal structure of the human p53 (2MEJ). Both molecules showed very similar docked poses to the one proposed by Fekry et al. (Figure 9). In both cases, the central polar part of the compounds was oriented close to the polar residues Arg280 and Asp281 at the base of the H2 helix, probably stabilized by forming water-mediated H bonds. The two long hydrophobic tails of both compounds fit in a complementary fashion into the deep groove between the H2 helix and the Ser240-P250 loop. To our delight, the C10 atom of the acyl chain of both ceramides was docked in close proximity to Ser240 and Ser241. These residues have been proven by the aforementioned research group, using MS-proteomics experiments, to participate in C_16_-ceramide binding to p53 [87]. Moreover, they used modified C_16_-ceramide with a diaziridine group to show that the ceramide C10 atom binds proximal to Ser240 and Ser241. Interestingly, a very similar orientation of C10 was noticed in the top-ranked docking poses of both ceramides **A** (**1**) and **B** (**2**).

## 3. Material and Methods

### 3.1. Plant Material

The red algae *Hypnea* *musciformis* was collected from Safaga at the Egyptian Red Sea in August 2017, air-dried, and stored at low temperature (−24 °C) until further processing. The plant was identified by Dr. Tarek Temraz of the Marine Science Department in the Faculty of Science at Suez Canal University in Ismailia, Egypt. A voucher specimen was deposited in the herbarium section of the Pharmacognosy Department of the Faculty of Pharmacy at Suez Canal University in Ismailia, Egypt under registration number SAA-130.

### 3.2. Metabolic Profiling

Metabolic profiling was performed using the liquid chromatography coupled with high-resolution electron spray ionization mass spectrometry technique (LC-HR-ESI-MS) as previously mentioned [20]. The obtained metabolites were described by comparison with several databases (e.g., DNP and METLIN).

### 3.3. Extraction and Isolation of Pure Compounds

The red algae *Hypnea musciformis* (430 g fresh weight) was freeze-dried, ground, and soaked in methanol (3 × 2 L) at room temperature. Then, the combined methanol extracts were evaporated under a vacuum, resulting in 32 g of green residue. The total methanol extract was then fractionated by partitioning with different solvents in increasing order of polarity several times to afford an *n*-hexane fraction (3.67 g), a chloroform fraction (2.33 g), an ethyl acetate fraction (7.43 g), an *n*-butanol fraction (3.07 g), and finally an aqueous fraction. The ethyl acetate fraction (Hp-EA, 7.43 g) was chromatographed with SiO_2_ column chromatography using hexane:EtOAc:methanol (80:20:0 up to 0:75:25) gradient elution, giving seven subfractions, Hp-EA-(1-7). Subfraction Hp-EA-4 (437 mg) was rechromatographed on silica gel using hexane:EtOAc:methanol (50:50:0 up to 0:90:10) gradient elution, giving five subfractions, Hp-EA-4-(1′-5′). One of the resultant subfractions, Hp-EA-4-2′, was applied to a sephadex LH-20 column eluted with CHCl_3_-MeOH (1:1), giving 3 subfractions, Hp-EA-4-2′-(1-3). Among them, ceramide **A** (**1**) (13 mg, white powder) was afforded from subfraction Hp-EA-4-2′-2. Ceramide **A** (**1**) was finally purified on an open ODS column using MeOH/H_2_O (8:2). Another subfraction, Hp-EA-4-2′-3, was subjected to silica gel column chromatography using EtOAc:methanol (95:5) to afford ceramide **B** (**2**) (15 mg, white powder) and ceramide **C** (**3**) (6 mg, white powder). Regarding the chloroform fraction (Hp-C, 2.33 g), it was chromatographed with SiO_2_ column chromatography using hexane:EtOAc (100:0 up to 25:75) gradient elution, giving 3 subfractions, Hp-C-(1-3). Subfraction Hp-C-1 (223 mg) was rechromatographed on silica gel using hexane:EtOAc (90:10 up to 70:30) gradient elution, giving two subfractions, Hp-C-1-(1′-2′). The first one, Hp-C-1-1′, afforded compound **4**, white powder 10 mg in weight, while the second subfraction (Hp-C-1-2′) was applied to a sephadex LH-20 column eluted with CHCl_3_-MeOH (1:1), giving compound **5** (12 mg, white powder) and compound **6** (13 mg, white powder).

### 3.4. In Vitro Cytotoxic Activity of Ceramides **A** (**1**), **B** (**2**), and **C** (**3**)

#### 3.4.1. In Vitro Cell Culture

The human breast adenocarcinoma (MCF-7) cell line was purchased from the American Type Culture Collection (ATCC HTB-22), Minnesota, USA). The tumor cell lines were maintained at the National Cancer Institute in Cairo, Egypt by serial subculturing. The cells were subcultured on RPMI 1640 medium supplemented by 1% penicillin/streptomycin and 10% fetal bovine serum [20].

#### 3.4.2. Sulforhodamine-B Assay

The cytotoxic activity of three isolated ceramides **A** (**1**), **B** (**2**), and **C** (**3**) was determined by a Sulforhodamine-B (SRB) assay. SRB is able to bind to the intracellular proteins, providing a sensitive index of the cellular protein content. It was assessed as previously described in detail [20]. The experiment was repeated 3 times, and the IC_50_ values (concentration that caused a 50% decrease in cell viability) were calculated. Doxorubicin was used as a positive control with the same concentration range.

### 3.5. In Vivo Study

Two ceramides were chosen for further investigation in vivo: ceramide **A** (**1**) and ceramide **B** (**2**). EAC is a spontaneous murine mammary adenocarcinoma model that has extensively been used as a study model of breast cancer [88]. The effect of these ceramides against EAC and their role in regulating vascular and apoptotic factors were tested.

#### 3.5.1. Tumor Induction

Human breast cancer cell line MCF-7 and EAC cells were purchased from the Tumor Biology Department at the National Cancer Institute of Cairo University. MCF-7 cells were cultured in Dulbecco’s Modified Eagle’s Medium (DMEM) supplemented with 10% heat-inactivated fetal bovine serum, 1% L-glutamine, HEPES buffer, and 50 µg/mL gentamycin. All cells were maintained at 37 °C in a fully humidified air atmosphere containing 5% CO_2_ and were subcultured twice a week. EAC served as the original tumor from which an ascites variant was obtained. EAC cells were suspended in normal saline (2.5 × 10^6^ cells/100 µL). Cells were counted by a hemocytometer under the microscope. The mice were inoculated intradermally with a 100-μL EAC suspension on the lower ventral side after shaving [89].

#### 3.5.2. Study Design

The study involved 48 Swiss albino mice obtained from the Egyptian Organization for Biological Products and Vaccines (Vacsera, Giza, Egypt) weighing 25–30 g. The mice were housed in plastic cages at a 25 °C temperature under a normal light/dark cycle, with water and food provided ad libitum. The mice were left for 1 week before the experiments to adjust. They were randomly separated into 6 groups (8 mice each). The first group was considered the normal group (negative control) and received Tween 80. The tumor cells were injected in all 5 other groups (groups 2–6). Group 2 was considered the EAC control group. Groups 3 and 4 received 1 mg/kg and 2 mg/kg of ceramide **A** (**1**), respectively. Groups 5 and 6 were treated by 1 mg/kg and 2 mg/kg of ceramide **B** (**2**), respectively. Ceramide **A** (**1**) and ceramide **B** (**2**) were dissolved in Tween 80 as a vehicle. The day of tumor cell injection was considered day zero (0). Groups 3–6 were treated daily from day 7 until day 21. The in vivo study was in agreement with the *Guide for the Care and Use of Laboratory Animals*. The study protocol was approved by the ethical committee of the Faculty of Pharmacy at Suez Canal University (201605PHDA1).

#### 3.5.3. Sample Collection

At the 21st day of EAC cell inoculation, the mice were anesthetized with thiopental sodium (50 mg/kg). Blood samples were collected from the orbital sinus (retro-orbital plexus). The blood samples were left to clot for 20 min, followed by separation of the serum by centrifugation at 2000× *g* for 15 min. The mice were sacrificed by cervical dislocation, and the tumor discs were separated, weighed, and separated into two portions; one portion of each tumor disc was fixed in 10% neutral buffered formalin for immunohistochemical investigations, while the other portion was kept at −80 °C for PCR analysis.

#### 3.5.4. Determination of Endothelial Growth Factor B (VEGF-B) and Tumor Necrosis Factor-α (TNF-α) in the Serum by ELISA

The serum samples were stored at −20 °C and used for determination of the levels of VEGF-B and TNF-α by ELISA kits ab213897 and ab181421, respectively (Abcam, Cambridge, UK), according to the manufacturer’s instructions. The serum levels of the liver function enzymes ALT and AST were assessed by colorimetric kits AL1031 and AS1061, respectively (Biodiagnostic, Giza, Egypt). Similarly, the serum levels of the kidney markers urea and creatinine were also determined calorimetrically via UR2110 and CR1250, respectively (Biodiagnostic, Giza, Egypt).

#### 3.5.5. Quantitative Real-Time PCR (q RT-PCT) for Assessment of the Expression of Midkine (MDK) in Tumor Tissue

The total RNA was isolated from the tumor tissue by an SV total RNA isolation system (Promega, Madison, WI, USA) according to the manufacturer’s instructions. The extracted RNA was stored at −80 °C. The concentration and purity of the isolated RNA were measured by a Nanodrop NA-1000 UV/Vis spectrophotomter (Thermo Fisher Scientific Inc., Wilmington, DE, USA). A GoTaq^®^ 1-Step RT-qPCR System (Promega, Madison, WI, USA) and the two primers, 5′-GTCAATCACGCCTGTCCTCT-3′ (forward) and 5′-CAAGTATCAGGGTGGGGAGA-3′ (reverse), were used for determination of the MDK expression. *β*-actin was marked as the housekeeping gene and was amplified using two primers: 5′-ACGGCCAGGTCATCACTATTG-3′ (forward) and 5′-CAAGAAGGAAGGCTGGAAAAGA-3′ (reverse). The 20-µL reaction mixture of each sample was composed of 4 µL of the RNA template, 1 μL of each of the forward and reverse primers, 0.4 μL of GoScript™ reverse transcriptase (RT) mix for 1-step RT-qPCR, 10 μL of GoTaq^®^ qPCR master mix, 0.31 μL of supplemental CXR reference dye, and 3.29 μL of nuclease-free water. The reaction was carried out in a StepOnePlus™ Real-Time PCR thermal cycler (Applied Biosystems, Waltham, MA, USA). The program was formed of reverse transcription at 37 °C for 15 min, inactivation of the reverse transcriptase enzyme, and initial denaturation at 10 min at 95 °C, followed by 40 cycles of denaturation at 95 °C for 10 s, annealing at 52 °C for 30 s, and extension at 72 °C for 30 s. The cycle threshold (Ct) of each reaction was recorded, and the ∆Ct was calculated against *β*-actin. The fold change of each sample was calculated to be 2^−∆∆Ct^.

#### 3.5.6. Immunohistochemical Assessment of the Expression of Apoptotic Markers in the Tumor Tissue

The tumor discs were fixed in 10% neutral buffered formalin overnight and then embedded in paraffin. Deparaffinization was performed by adding xylene and ethyl alcohol in decreasing concentrations from 100% to 70%. Antigen retrieval was performed according to the Tris/EDTA buffer (pH = 9) antigen retrieval protocol. The EnVision™ FLEX HRP-labeled high-pH method was used for staining according to the manufacturer’s protocol (Dako, Glostrup, Denmark). The primary polyclonal antibodies for p53, Bax, and caspase 3 (Bioss Inc., Woburn, MA, USA) were diluted in PBS (normal phosphate buffered saline) at a ratio of 1:250. Finally, Mayer’s hematoxylin was used for counterstaining.

ImageJ was used for the semiquantitative analysis of the immunohistochemical reactions. The images were captured by an optical microscope with a 40× objective (Optika B-352A, OPTICA, Via Rigla, Italy) coupled to a camera (HDCE30C) using its software and quantified using the ImageJ MacBiophotonics (National Institutes of Health, Bethesda, MD, USA) software package developed by McMaster University (Hamilton, Ontario, Canada). The expressions of p53, Bax, and caspase 3 were all assessed, and the percentages of stained areas were calculated using the color deconvolution plugin.

#### 3.5.7. Statistical Analysis

The values of the determined parameters were expressed as mean ± standard deviation (SD). Comparisons were performed by one-way ANOVA followed by a Bonferroni post hoc test for multiple comparisons. Differences at *p* < 005 were considered statistically significant.

### 3.6. Molecular Modeling Study

Molecular Operating Environment (MOE) was used to dock both ceramides **A** (**1**) and **B** (**2**) into the transcription factor P53 [90]. First, the crystal structures of P53 (2MEJ) were imported into the MOE graphical interface, and the protein was then prepared for docking using the default parameters of the Protein Preparation module and the Protonate 3D tool. Ligands were also sketched using MOE and minimized with the MMFF94 force field to a gradient of 0.001 kcal/mol·Å^2^. They were then docked into the putative binding site of the P53 protein using the induced-fit protocol and the default parameters of the MOE Dock module with the Triangle Matcher method. Residues Ser240 and Ser241 were used to specify the binding site as reported in the literature. The default values of 30 docked structures for each ligand were used. Poses were arranged according to the docking scores and inspected visually.

## 4. Conclusions

From the methanolic extract of the Red Sea red algae *Hypnea musciformis*, two new ceramides and another first reported one were isolated. Moreover, other chemical metabolites were detected by using the LC-ESI-HRMS technique. Both new metabolites, ceramides **A** (**1**) and **B** (**2**), exhibited significant in vitro cytotoxic activity against the human breast adenocarcinoma (MCF-7) cell line. The activity of ceramides **A** (**1**) and **B** (**2**) was investigated in an EAC mouse model, where both ceramides at doses of 1 and 2 mg/kg significantly decreased the tumor size, serum levels of VEGF-B and TNF-α, and expression of the biomarker midkine growth factor in the tumor tissue, with significant upregulation of the apoptotic factors p53, Bax, and caspase-3. Ceramide **B** (**2**), at a therapeutic dose of 2 mg/kg, showed the most potent antiangiogenic activity and the highest expression of the investigated apoptotic factors. Molecular docking suggested the interaction of these ceramides with the p53 transcription factor, leading to its accumulation and activation of the downstream apoptotic pathways. The current study introduced two potentially effective anti-cancer ceramides isolated from the Red Sea algae *Hypnea musciformis*, which exhibited antiangiogenic and apoptotic effects in the experimentally induced mammary tumor. The major limitation of this work is that the effects of the isolated ceramides were not investigated on healthy cell lines, suggesting that further research is required to evaluate the toxicity of those compounds and to determine their therapeutic indices.

## Figures and Tables

**Table 1:** Metabolic profiling (LC-ESI-HRMS) of methanolic crude extract of *Hypnea* sp.

No.	Polarity Mode	Ret. Time (min)	*m*/*z*	MZmine ID	Detected Mass	Expected Mass	Mass Error (ppm)	Name	Source	Ref.
1	Positive	12.70	399.3255	292	398.3182	398.3185	−0.75	Diketosteroid	Red alga *Hypnea musciformis*	[10]
2	Positive	12.70	399.3255	292	398.3182	398.3185	−0.75	6α-Hydroxy-cholesta-4,22-diene-3-one	Red alga*Hypnea musciformis*	[11]
3	Positive	12.70	399.3255	292	398.3182	398.3185	−0.75	Cholesta-5,22-diene-3*β* -ol-7-one	Red alga *Hypnea flagelliformis*	[13]
4	Positive	12.70	399.3255	292	398.3182	398.3185	−0.75	5*β* -Cholest-3-ene-7,11-dione	Red alga*Hypnea musciformis*	[10]
5	Positive	12.49	385.3465	326	384.3392	384.3392	0.00	Cholesta-5,22-dien-3*β*-ol	Red alga *Hypnea flagelliformis*	[13]
6	Positive	12.49	385.3465	326	384.3392	384.3392	0.00	22-Dehydrocholesterol	Red alga *Hypnea flagelliformis*	[13]
7	Positive	8.44	318.3005	108	317.2932	317.2930	0.63	Phytosphingosine	Fungi	[25]
8	Negative	6.33	349.2013	1839	350.2085	350.2093	−2.28	PGE2, Prostaglandin-E2	Red alga *Gracilaria verrucosa*	[24]
9	Positive	6.99	333.2064	1326	332.1991	332.1988	0.90	Ptilodene	Red alga*Ptilota filicina*	[21]
10	Negative	6.78	331.1914	1751	332.1987	332.1988	−0.30	Agardhilactone	Red alga *Agardhiella subulata*	[22]

**Table 2:** The ^1^H (400 MHz) and ^13^C (100 MHz) NMR spectroscopic data of isolated compounds **1**, **2**, and **3** (*δ* in ppm, *J* in Hz).

Compound 1 (CDCl_3_)			Compound 2 (C_5_D_5_N)			Compound 3 (C_5_D_5_N)		
No.	*_δ_*_H_ (ppm)	*_δ_*_C_ (ppm)	No.	*_δ_*_H_ (ppm)	*_δ_*_C_ (ppm)	No.	*_δ_*_H_ (ppm)	*_δ_*_C_ (ppm)
1	Ha: 3.75 (m)Hb: 4.08 (m)	61.9	1	Ha: 4.50 (m)Hb: 4.44 (m)	61.7	1	4.48 (m)	62.1
2	3.9 (m)	54.4	2	5.11 (m)	52.6	2	5.05 (m)	53.7
3	4.08 (m)	74.0	3	4.35 (m)	76.4	3	4.38 (m)	76.6
4	5.51 (m)	129	4	4.28 (m)	72.7	4	4.27 (m)	73.0
5	5.67 (m)	134.1	5	1.92 (m)	33.7	5	1.93 (m)	33.8
6	2.1 (m)	32.5	6	1.7 (m)	25.5	6	2.22 (m)	26.5
7	2.07 (m)	27.5	7–16	1.22–1.29 (m)	29.6	7–16	1.25 (m)	29.5–29.9
8	5.08 (br t, *J* = 12)	123.1	17	1.34 (m)	31.8	17	1.68 (m)	32.0
9	--	136.3	18	0.85 (3H, m, *J* = 8)	13.9	18	1.36 (m)	22.8
10	1.95 (t, *J* = 8)	39.7	1′	---	175.1	19	0.85 (t, *J* = 8)	14.1
11–19	1.12–1.38 (m)	29.2–29.7	2′	4.61 (m)	72.1	1′	---	173.4
20	1.12–1.32 (m)	31.9	3′	2.03 (m)	35.7	2′	2.45 (t, *J* = 8)	36.8
21	1.12–1.32 (m)	22.6	4′	2.25 (m)	32.1	3′	1.81 (m)	26.3
22	0.88 (t, *J* = 8.8)	14.0	5′	1.92 (m)	27.5	4′-13′	1.25 (m)	29.5–29.9
23	1.57 (s)	15.9	6′	1.7 (m)	22.5	14′	1.68 (m)	32.0
1′	---	175.7	7′	1.25 (m)	22.5	15′	1.36 (m)	22.8
2′	4.23 (t)	72.5	8′	2.08 (m)	26.4	16′	0.85 (t, *J* = 8)	14.1
3′	1.76 (m)	34.5	9′	5.47 (m)	130.0	NH	8.50 (d, *J* = 8)	---
4′	1.40 (m)	25.1	10′	5.47 (m)	130.0			
5′-19′	1.12–1.38 (m)	29.3–29.7	11′	2.08 (m)	26.4			
20′	1.12–1.38 (m)	31.9	12′-16′	1.22–1.29 (m)	29. 6			
21′	1.12–1.38 (m)	22.6	17′	1.34 (m)	31.8			
22′	0.88 (t, *J* = 8.8)	14.0	18′	0.85 (m, *J* = 8)	13.9			
NH	7.27 (d, *J* = 12)	---	NH	8.58 (d, *J* = 8)	---			

**Table 3:** IC_50_ values (µM) of ceramides **A** (**1**), **B** (**2**), and **C** (**3**) against human breast cancer MCF-7 cell line using doxorubicin as a positive control.

Compound	Human Breast MCF-7 Cell Line IC_50_ (µM)
Ceramide **A** (**1**)	11.07 ± 0.23 *
Ceramide **B** (**2**)	10.17 ± 0.15 *
Ceramide **C** (**3**)	19.34 ± 0.46 *
Doxorubicin	8.65 ± 0.03

* Significantly different compared with positive control doxorubicin. Each data point represents the mean ± SD of three independent experiments (significant differences at *p* < 0.05).

## Data Availability

Data are contained within the article and the Supplementary Materials.

## Supplementary Materials

The following are available online at https://www.mdpi.com/article/10.3390/md20010063/s1, Figures S1 and S2: LC-ESI-HRMS chromatogram of crude extract of *Hypnea* *musciformis* (positive and negative modes); Figure S3: ESI-HRMS chromatogram of ceramide **A** (**1**); Figures S4 and S5: ^1^H NMR and ^13^C NMR spectra of ceramide **A** (**1**); Figure S6: ESI-HRMS chromatogram of fatty acid methyl ester of ceramide **A** (**1**); Figure S7: ESI-HRMS chromatogram of ceramide **B** (**2**); Figures S8 and S9: ^1^H NMR and ^13^C NMR spectra of ceramide **B** (**2**); Figure S10: ESI-HRMS chromatogram of fatty acid methyl ester of ceramide **B** (**2**); Figure S11: GC-MS analysis of fatty acid methyl ester of ceramide **B** (**2**); Figure S12: ESI-HRMS chromatogram of ceramide **C** (**3**); Figures S13 and S14: ^1^H NMR and ^13^C NMR spectra of ceramide **C** (**3**); Figure S15: ESI-HRMS chromatogram of fatty acid methyl ester of ceramide **C** (**3**); Figure S16: ESI-HRMS chromatogram of compound **4**; Figures S17 and S18: ^1^H NMR and ^13^C NMR spectra of compound **4**; Figure S19: ESI-HRMS chromatogram of compound **5**; Figures S20 and S21: ^1^H NMR and ^13^C NMR spectra of compound **5**; Figure S22: ESI-HRMS chromatogram of compound **6**; Figure S23: ^1^H NMR spectra of compound **6**; Table S1: Liver enzymes and kidney markers in the study groups.
