# Supplementary material for: Anticancer Effects of New Ceramides Isolated from the Red Sea Red Algae Hypnea musciformis in a Model of Ehrlich Ascites Carcinoma: LC-HRMS Analysis Profile and Molecular Modeling
Source: Mar Drugs. 2022 Jan 10;20(1):63. doi: 10.3390/md20010063 (PMC8778197; doi:10.3390/md20010063)
Supplement: Supplementary file 1 [file marinedrugs-20-00063-s001.zip › marinedrugs-1543313-supplementary.pdf]

Anticancer Effects of New Ceramides Isolated from the Red Sea Red Algae *Hypnea musciformis* In a Model of Ehrlich Ascites Carcinoma: LC-HRMS Analysis Profile and Molecular Modeling

Additional Experimental Detail

No.		Page
Figure S1	LC-ESI-HRMS chromatogram of crude extract of *Hypnea musciformis* (positive mode).	2
Figure S2	LC-ESI-HRMS chromatogram of crude extract of *Hypnea musciformis* (negative mode).	3
Figure S3	ESI-HRMS chromatogram of ceramide **A** (**1**).	4
Figure S4	^{1}H NMR spectra of ceramide **A** (**1**).	5
Figure S5	^{13}C NMR spectra of ceramide **A** (**1**).	6
Figure S6	ESI-HRMS chromatogram of fatty acid methyl ester of ceramide **A** (**1**).	7
Figure S7	ESI-HRMS chromatogram of ceramide **B** (**2**).	8
Figure S8	^{1}H NMR spectra of ceramide **B** (**2**).	9
Figure S9	^{13}C NMR spectra of ceramide **B** (**2**).	10
Figure S10	ESI-HRMS chromatogram of fatty acid methyl ester of ceramide **B** (**2**).	11
Figure S11	GC-MS analysis of fatty acid methyl ester of ceramide **B** (**2**).	12
Figure S12	ESI-HRMS chromatogram of ceramide **C** (**3**).	13
Figure S13	^{1}H NMR spectra of ceramide **C** (**3**).	14
Figure S14	^{13}C NMR spectra of ceramide **C** (**3**).	15
Figure S15	ESI-HRMS chromatogram of fatty acid methyl ester of ceramide **C** (**3**).	16
Figure S16	ESI-HRMS chromatogram of compound **4**.	17
Figure S17	^{1}H NMR spectra of compound **4**.	18
Figure S18	^{13}C NMR spectra of compound **4**.	19
Figure S19	ESI-HRMS chromatogram of compound **5**.	20
Figure S20	^{1}H NMR spectra of compound **5**.	21
Figure S21	^{13}C NMR spectra of compound **5**.	22
Figure S22	ESI-HRMS chromatogram of compound **6**.	23
Figure S23	^{1}H NMR spectra of compound **6**.	24
Table S1	Liver enzymes and kidney markers in the study groups.	25

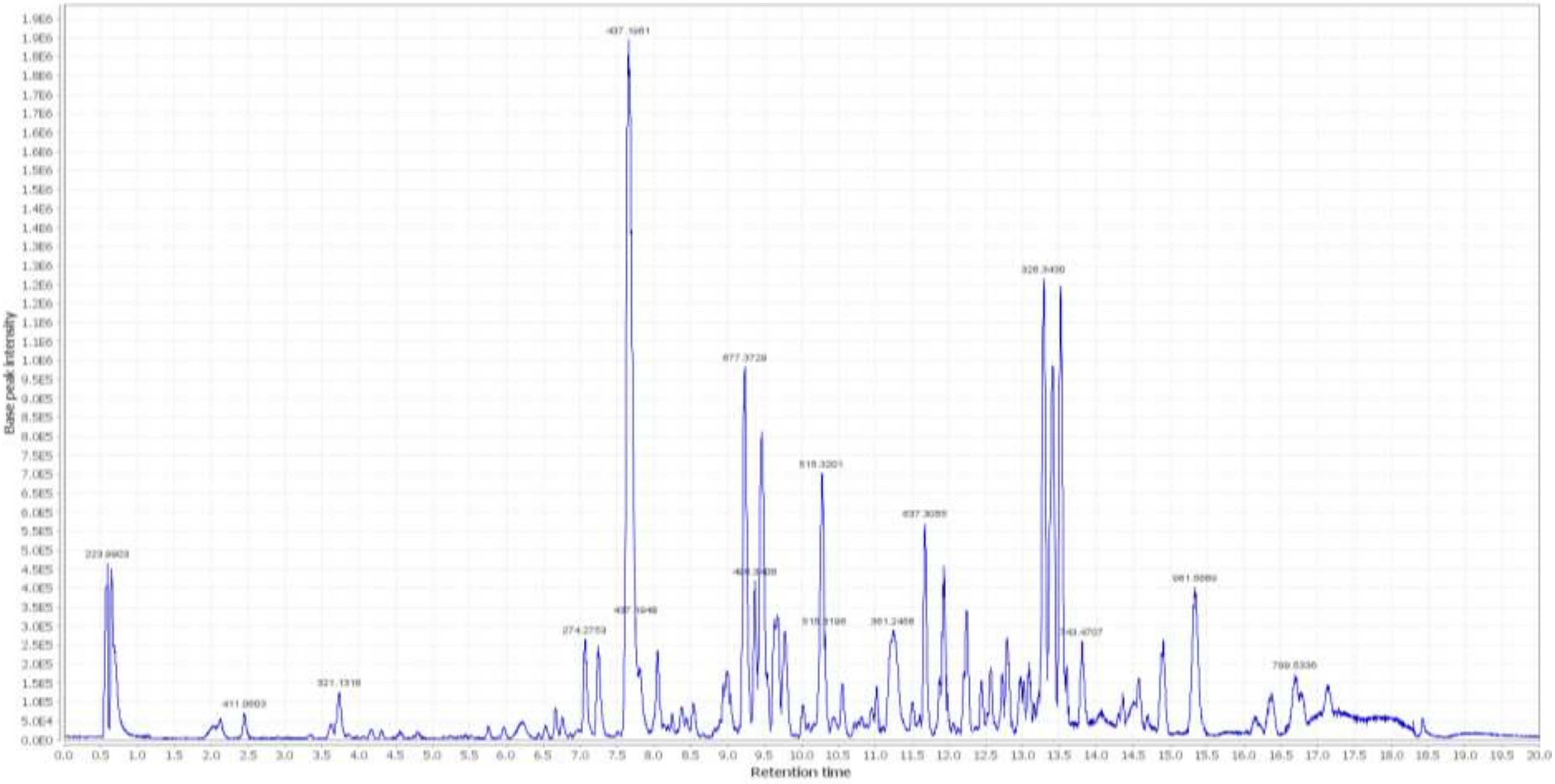

Figure S1. LC-ESI-HRMS chromatogram of crude extract of *Hypnea musciformis* (positive mode).

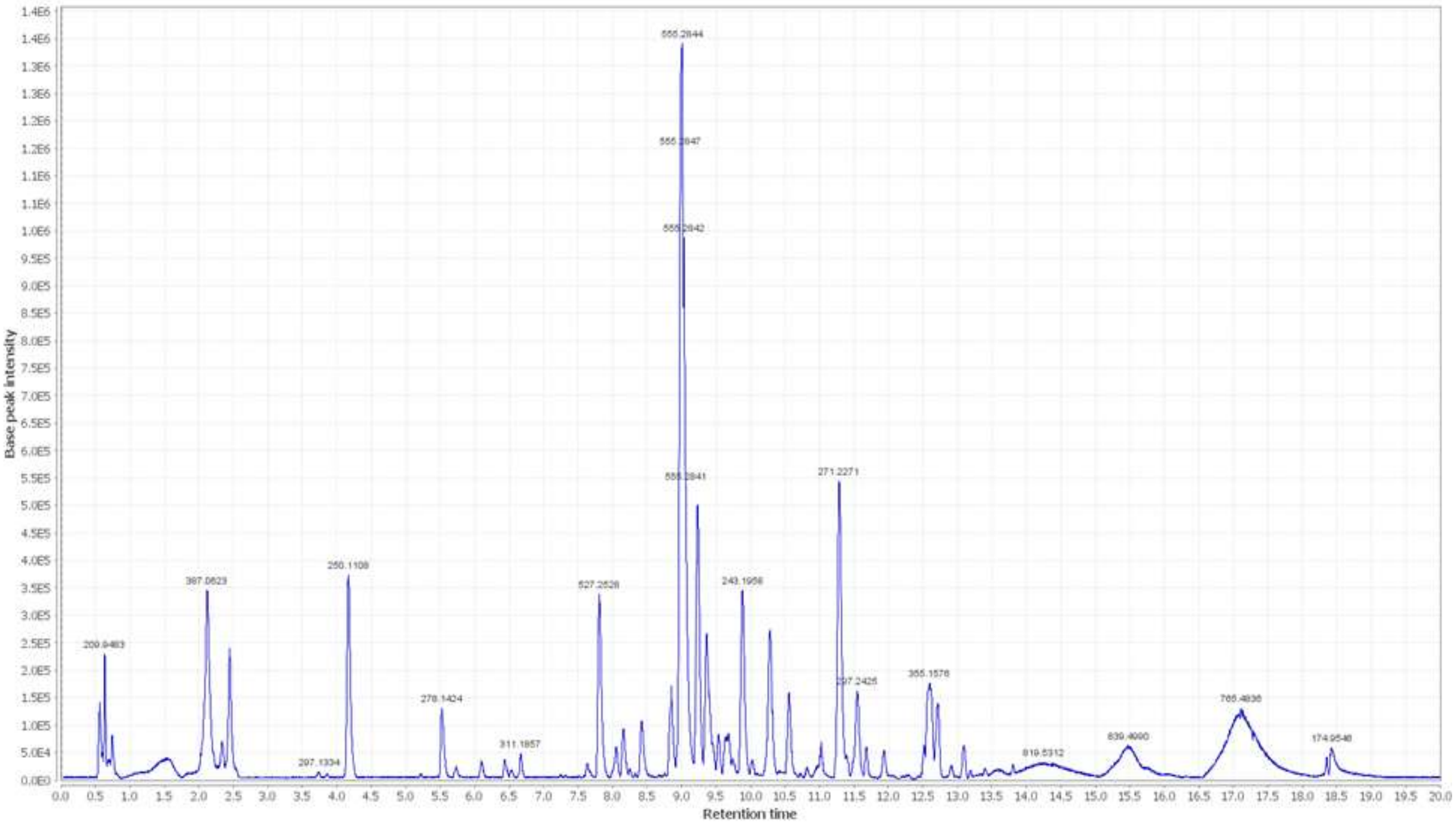

Figure S2. LC-ESI-HRMS chromatogram of crude extract of *Hypnea musciformis* (negative mode).

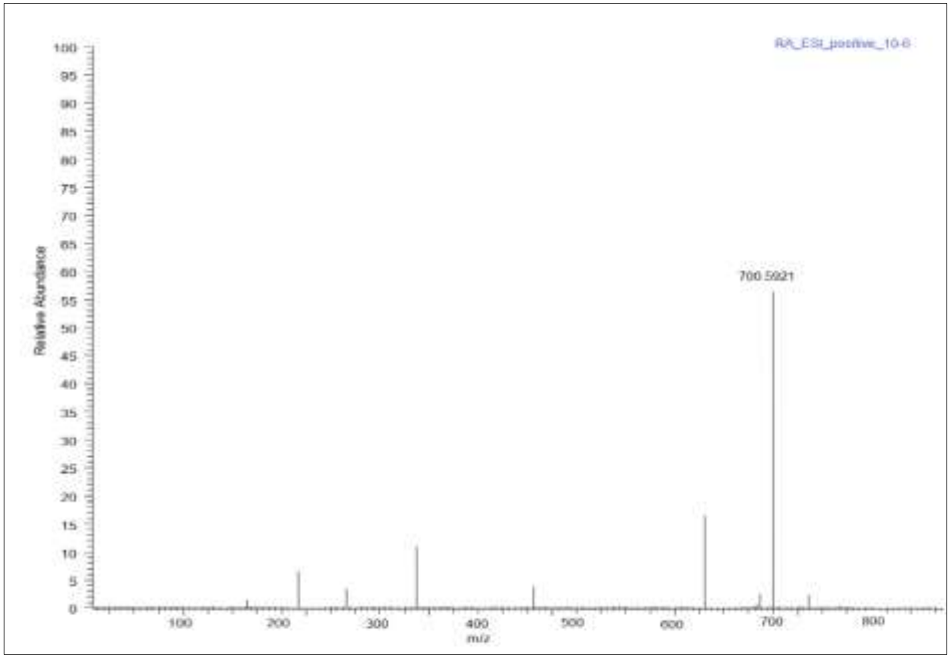

Figure S3. ESI-HRMS chromatogram of ceramide **A** (**1**)

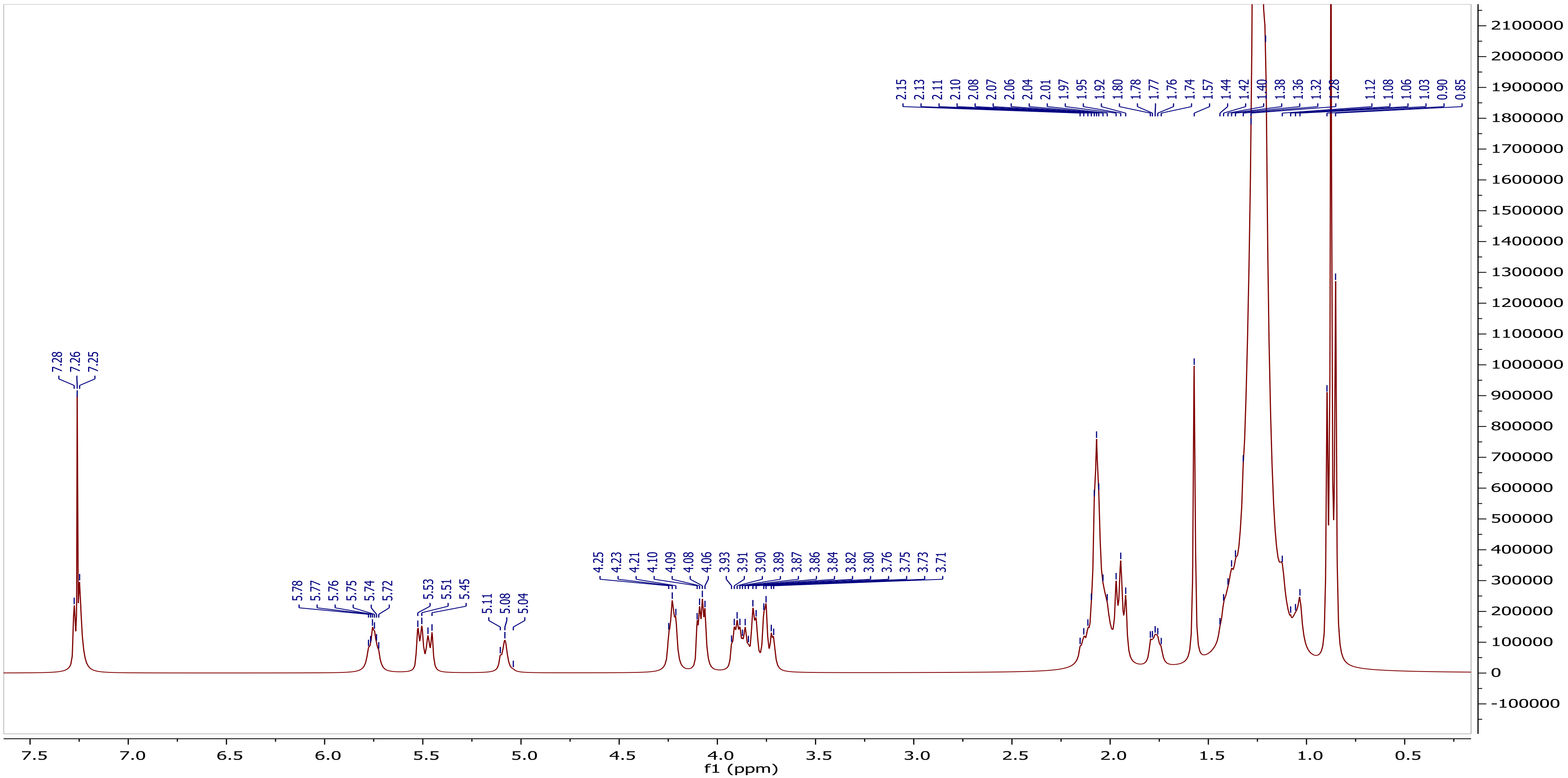

Figure S4. ^{1}H NMR spectra of ceramide **A** (**1**)

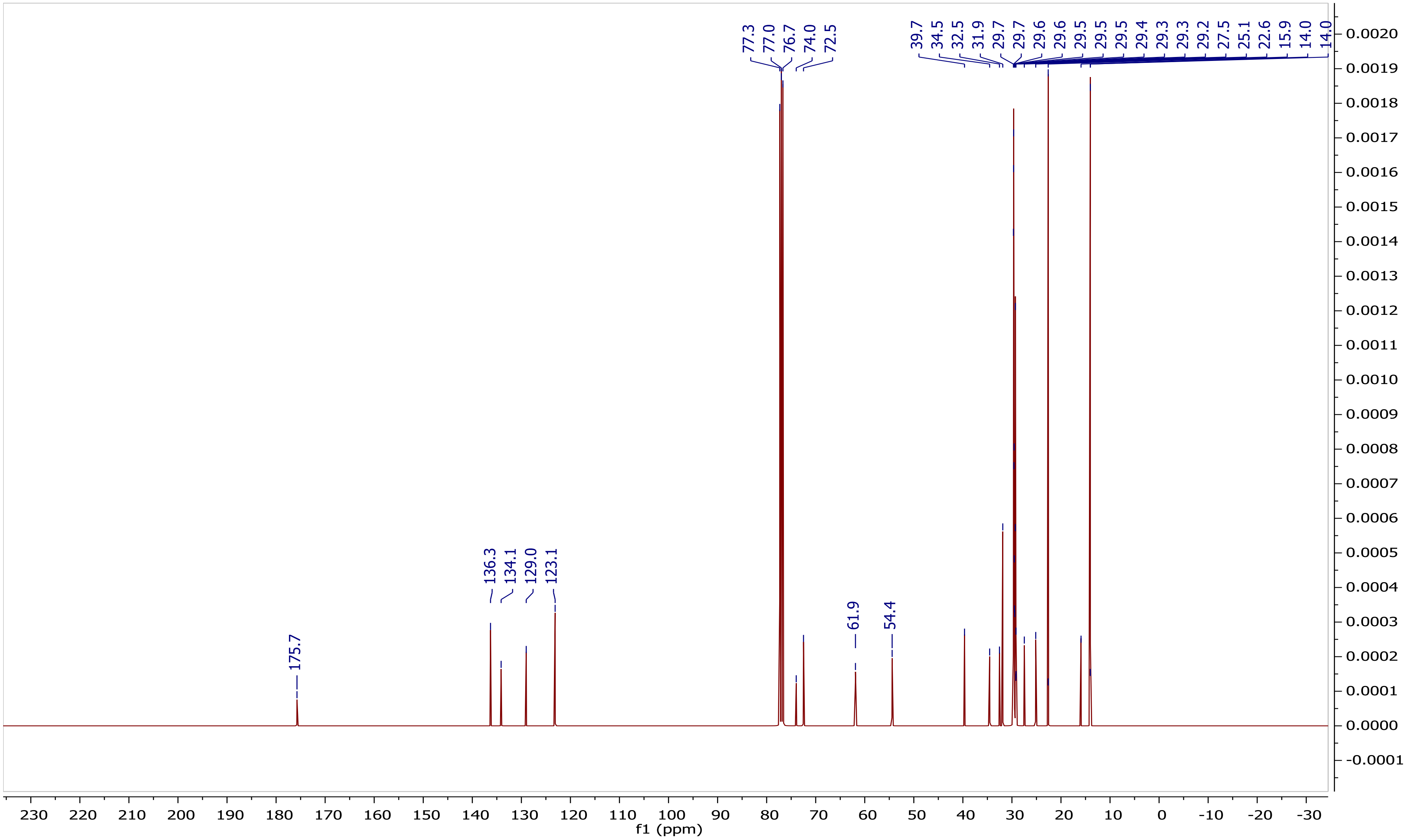

Figure S5. ^{13}C NMR spectra of ceramide **A** (**1**)

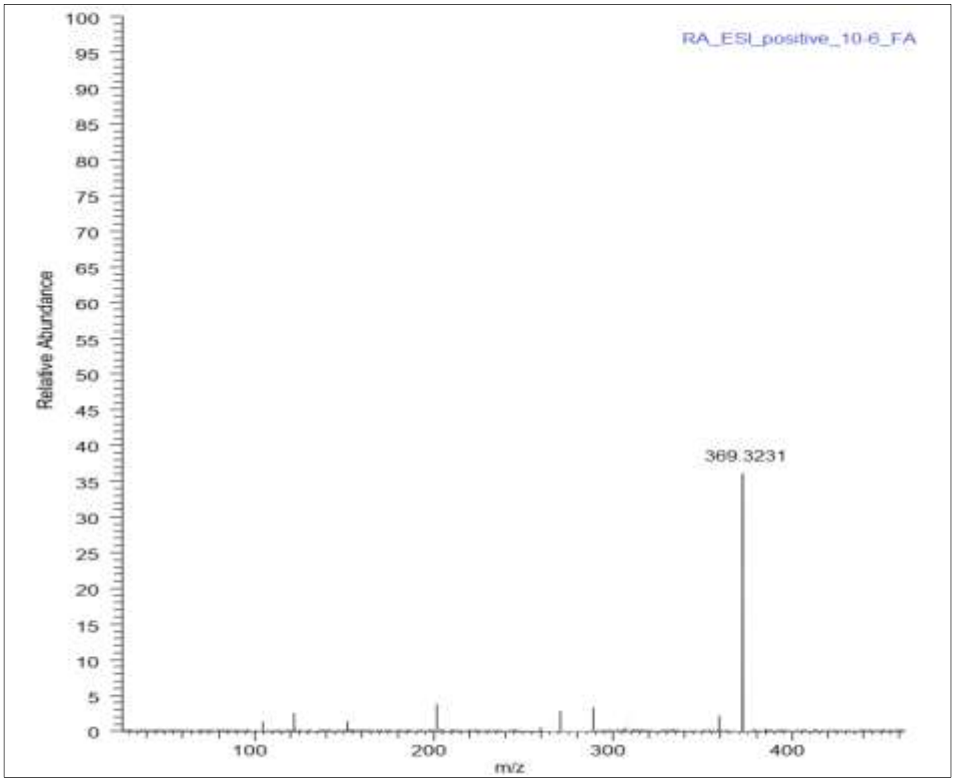

Figure S6. ESI-HRMS chromatogram of fatty acid methyl ester of ceramide **A** (**1**)

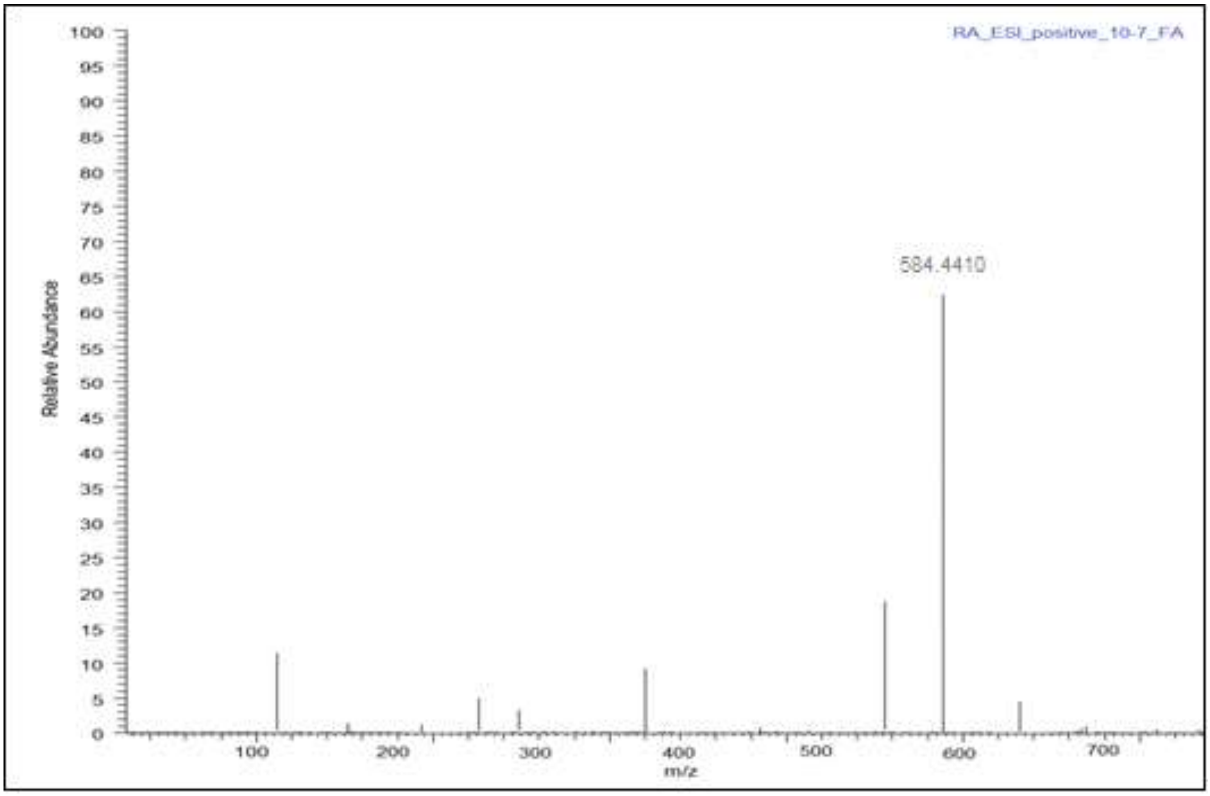

Figure S7. ESI-HRMS chromatogram of ceramide **B** (**2**)

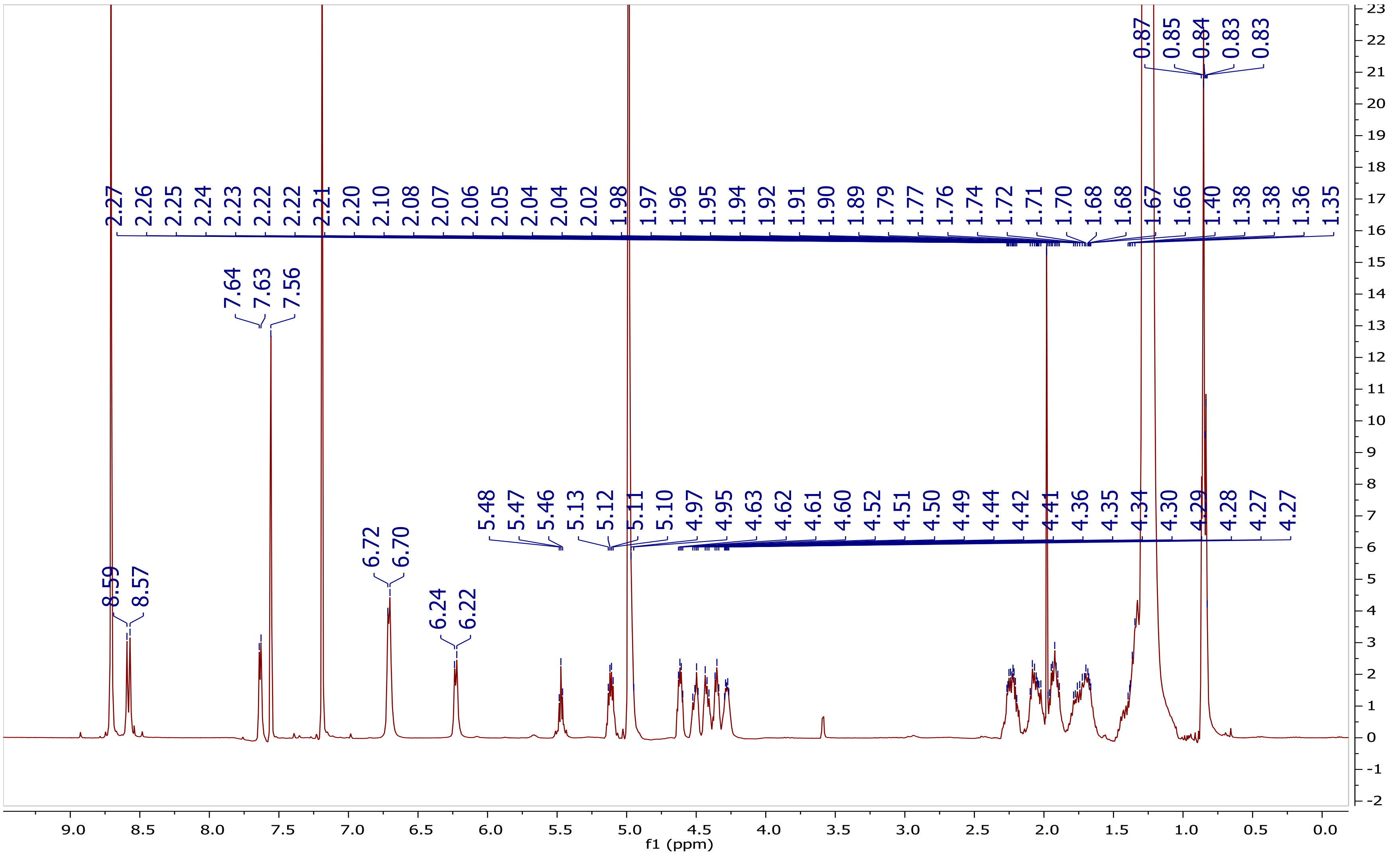

Figure S8. ^{1}H NMR spectra of ceramide **B** (**2**)

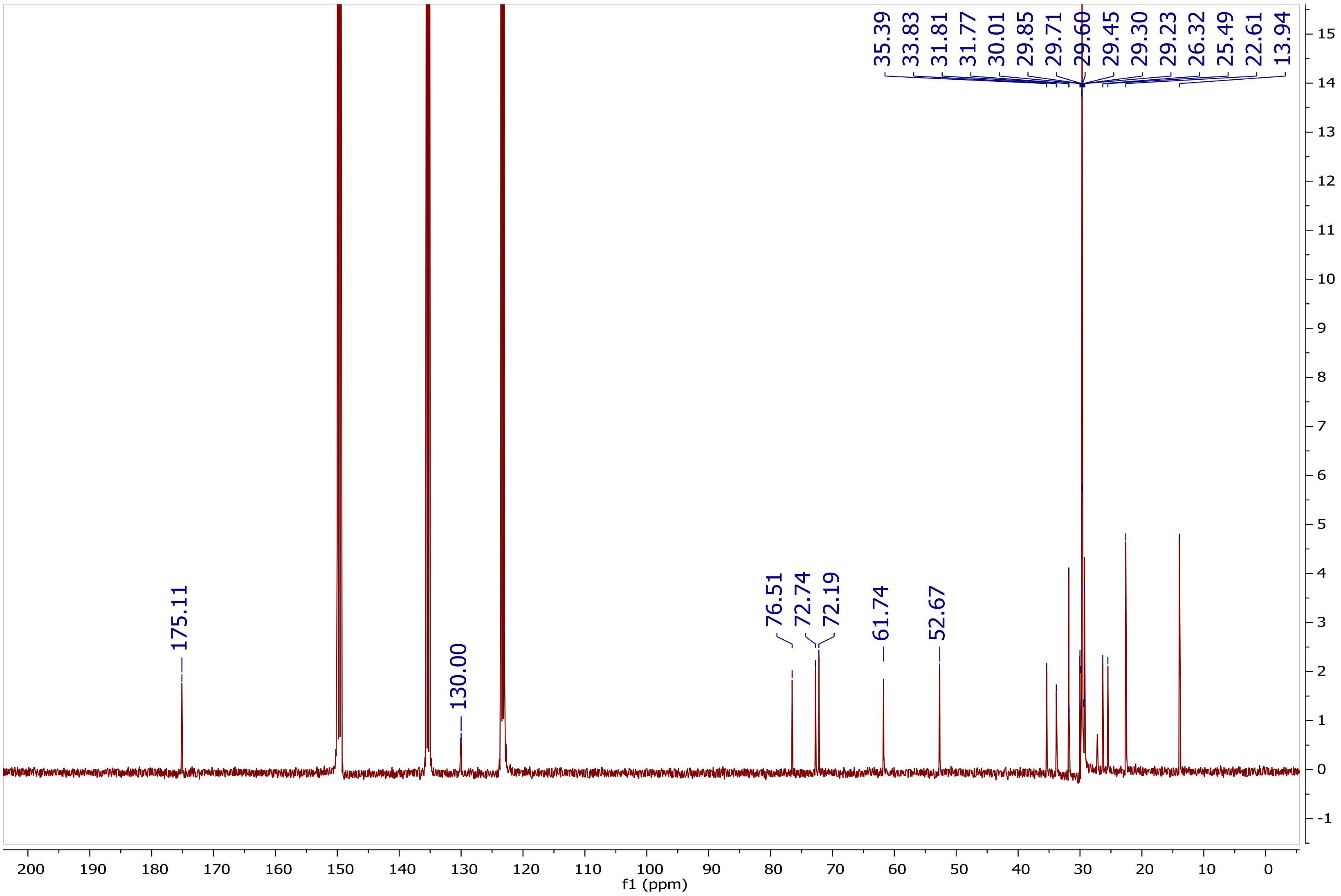

Figure S9. ^{13}C NMR spectra of ceramide **B** (**2**)

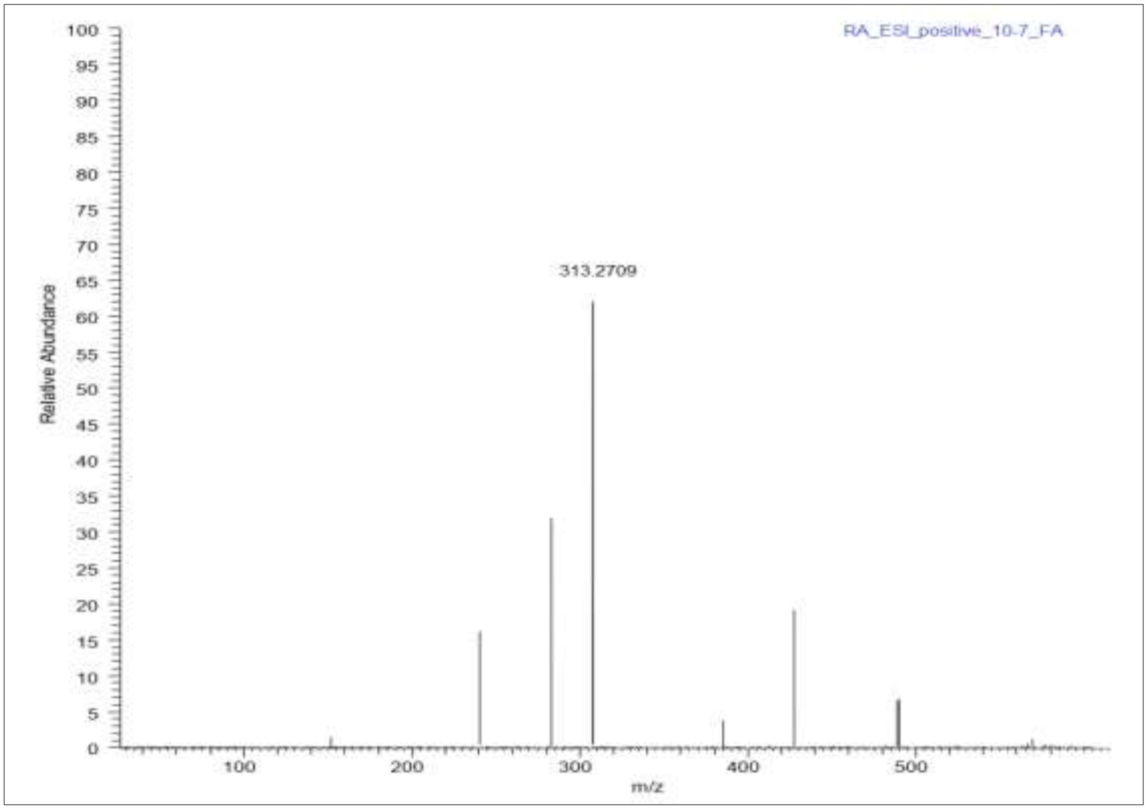

Figure S10. ESI-HRMS chromatogram of fatty acid methyl ester of ceramide **B** (**2**)

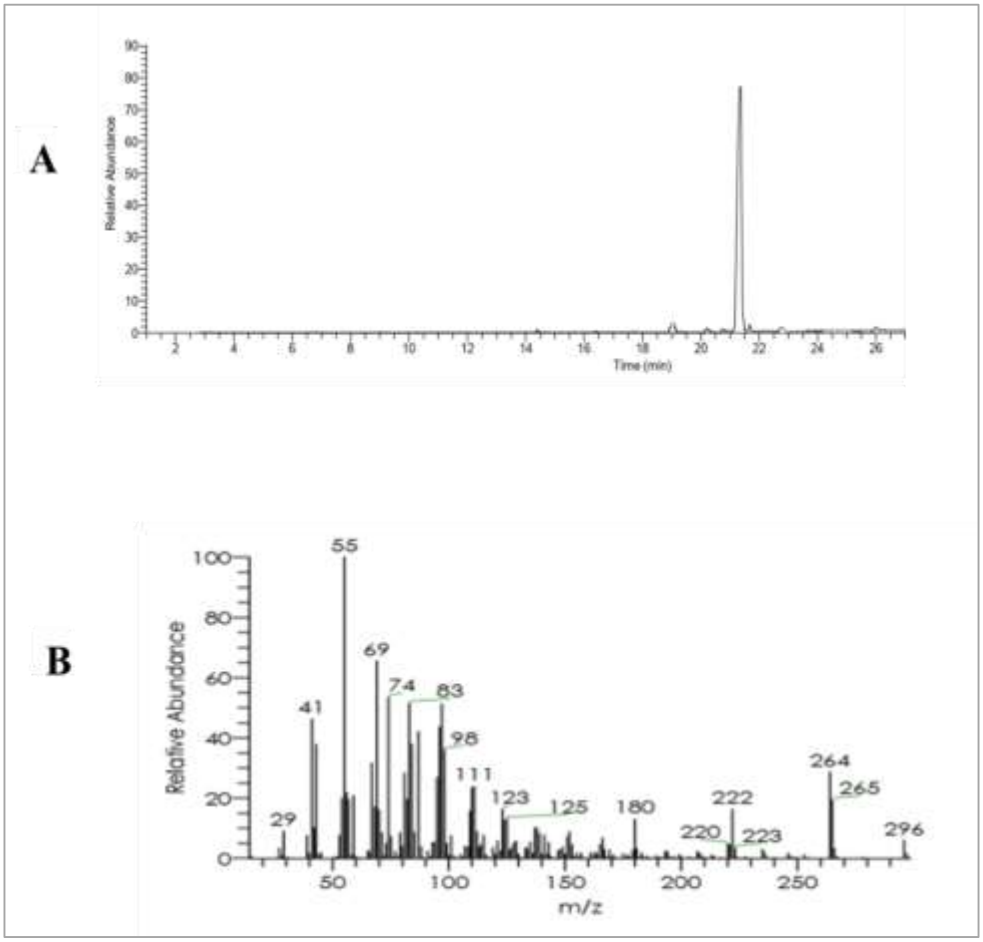

Figure S11. GC-MS analysis, GC-MS chromatogram (**A**) and fragmentation pattern (**B**), of fatty acid methyl ester of ceramide **B** (**2**)

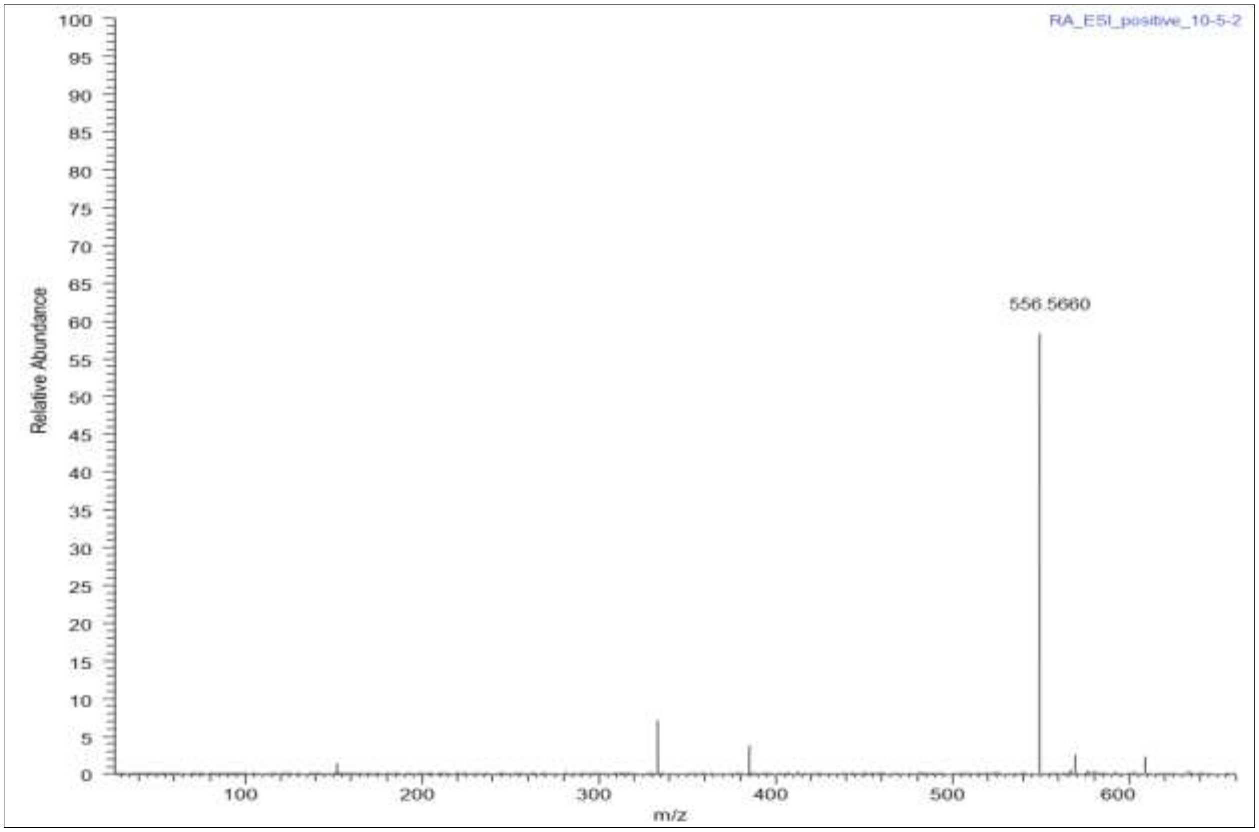

Figure S12. ESI-HRMS chromatogram of ceramide **C** (**3**)

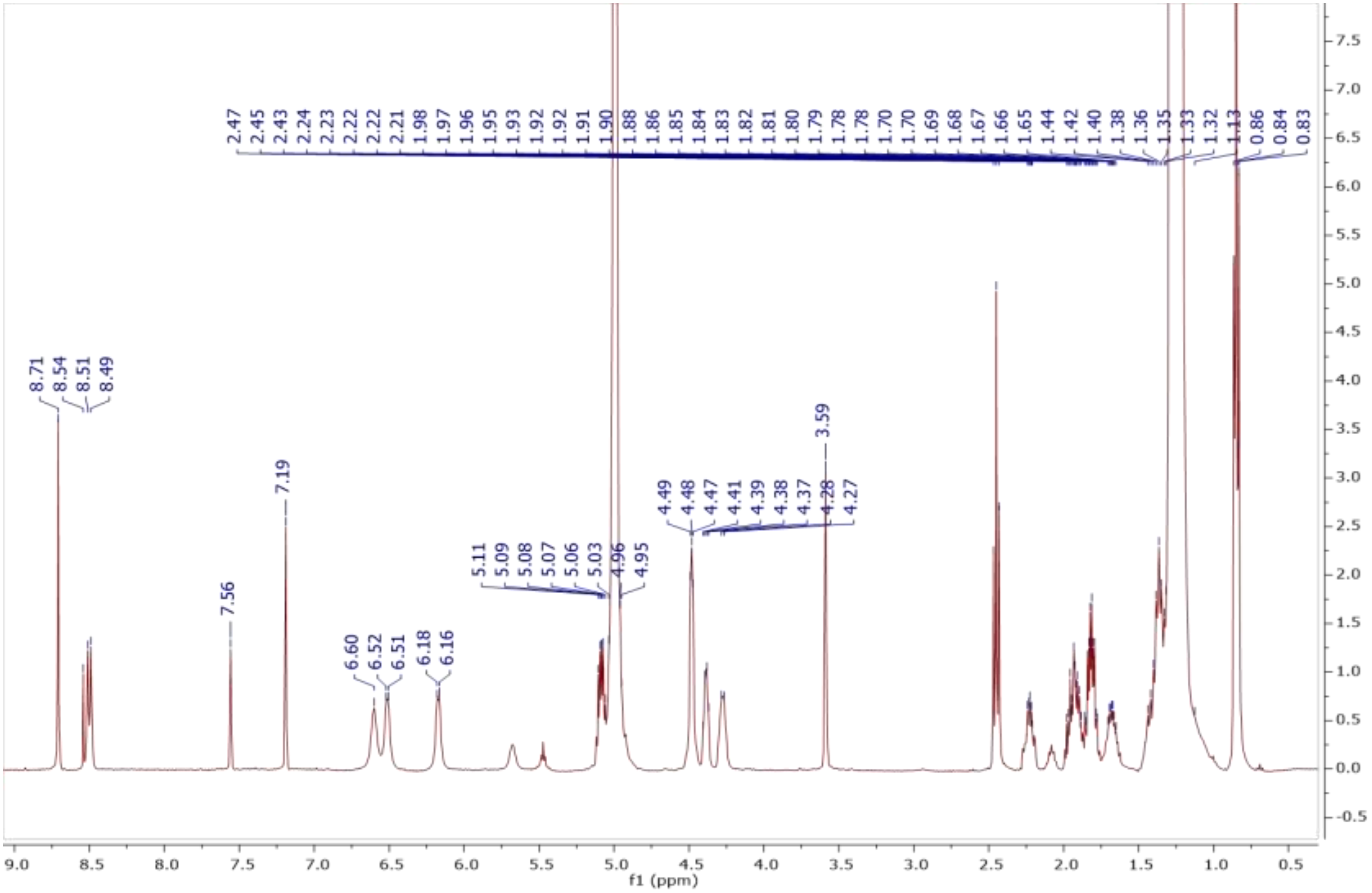

Figure S13. ^{1}H NMR spectra of ceramide **C** (**3**)

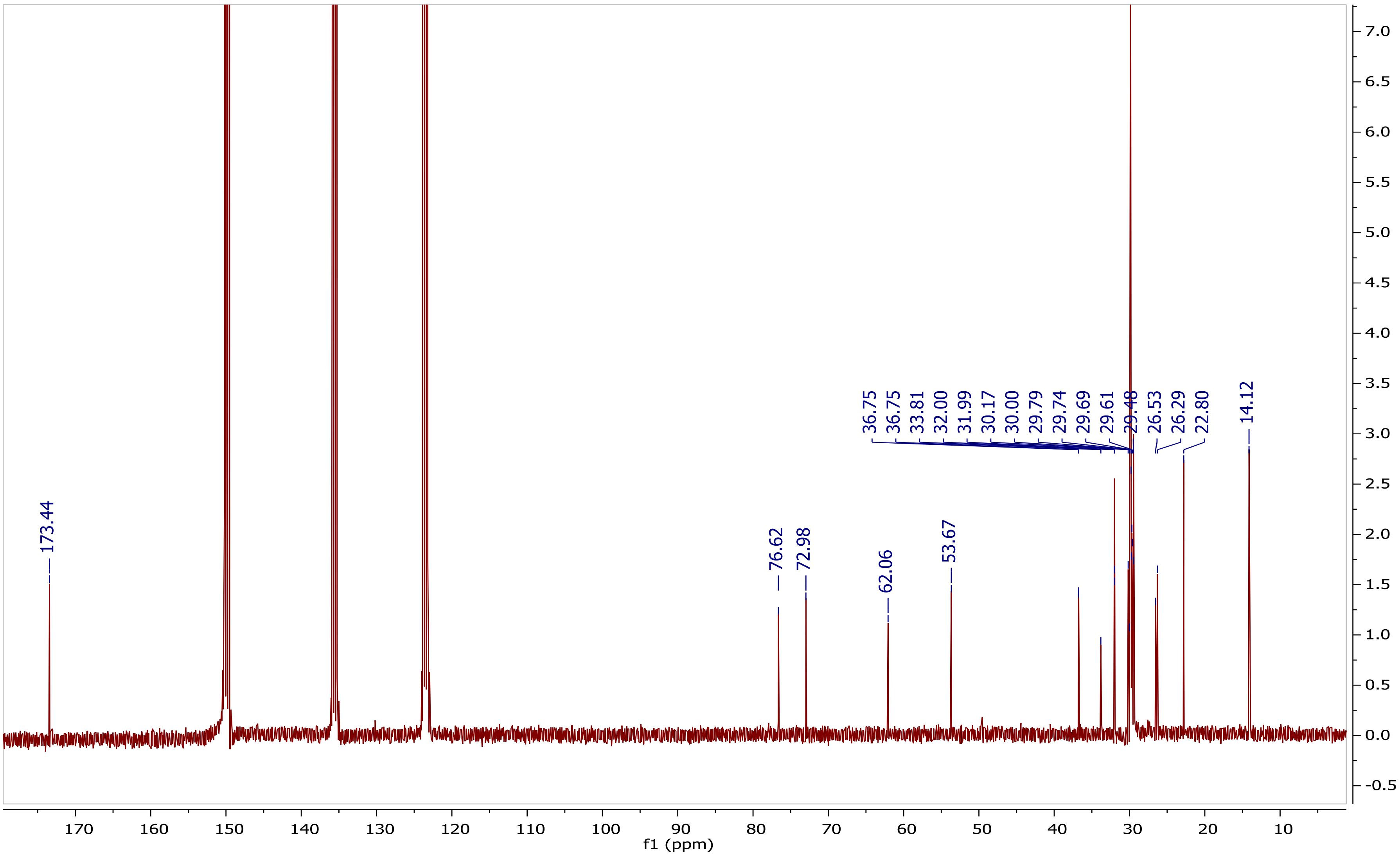

Figure S14. ^{13}C NMR spectra of ceramide **C** (**3**)

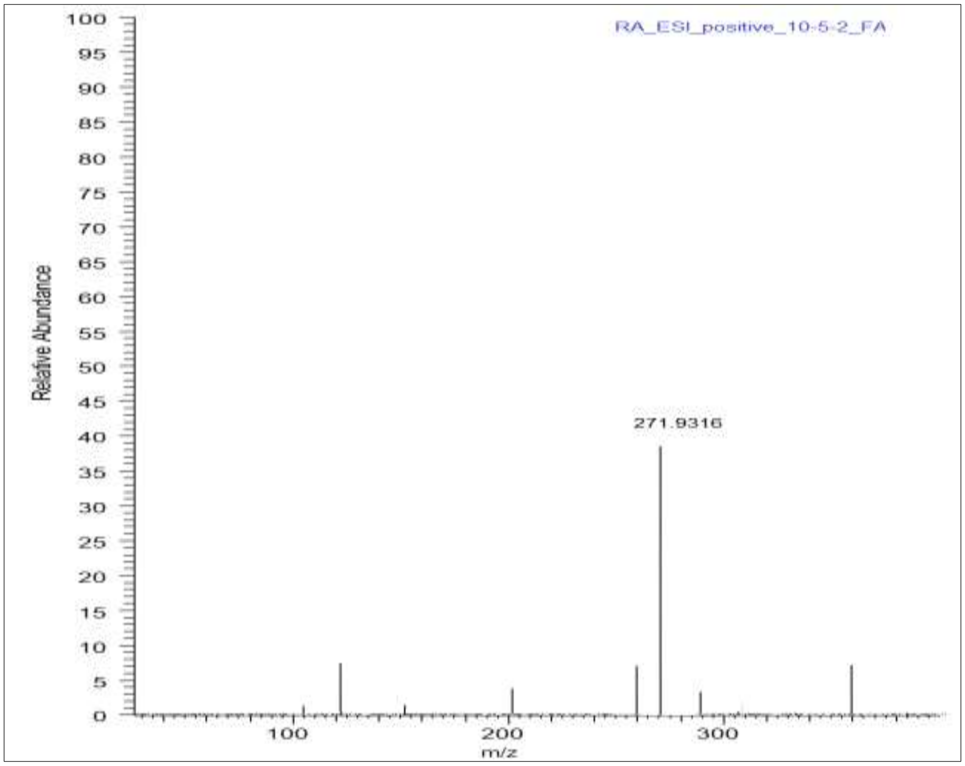

Figure S15. ESI-HRMS chromatogram of fatty acid methyl ester of ceramide **C** (**3**)

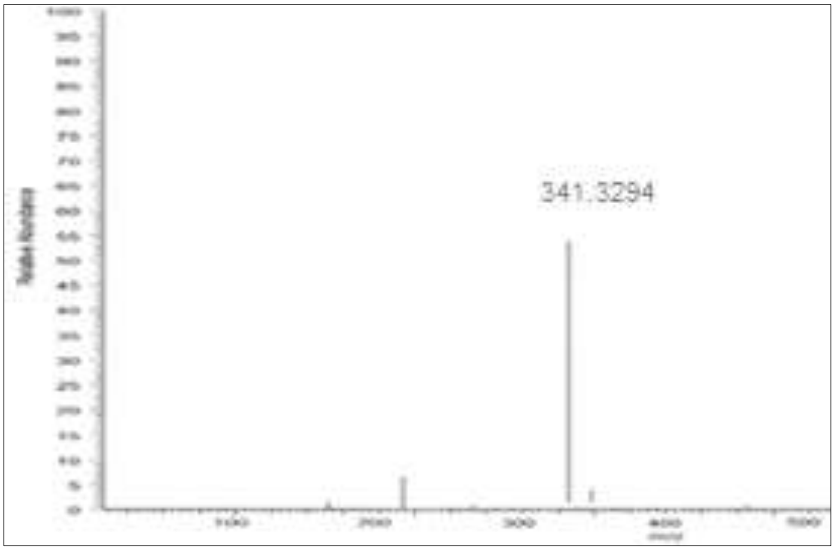

Figure S16. ESI-HRMS chromatogram of compound **4**.

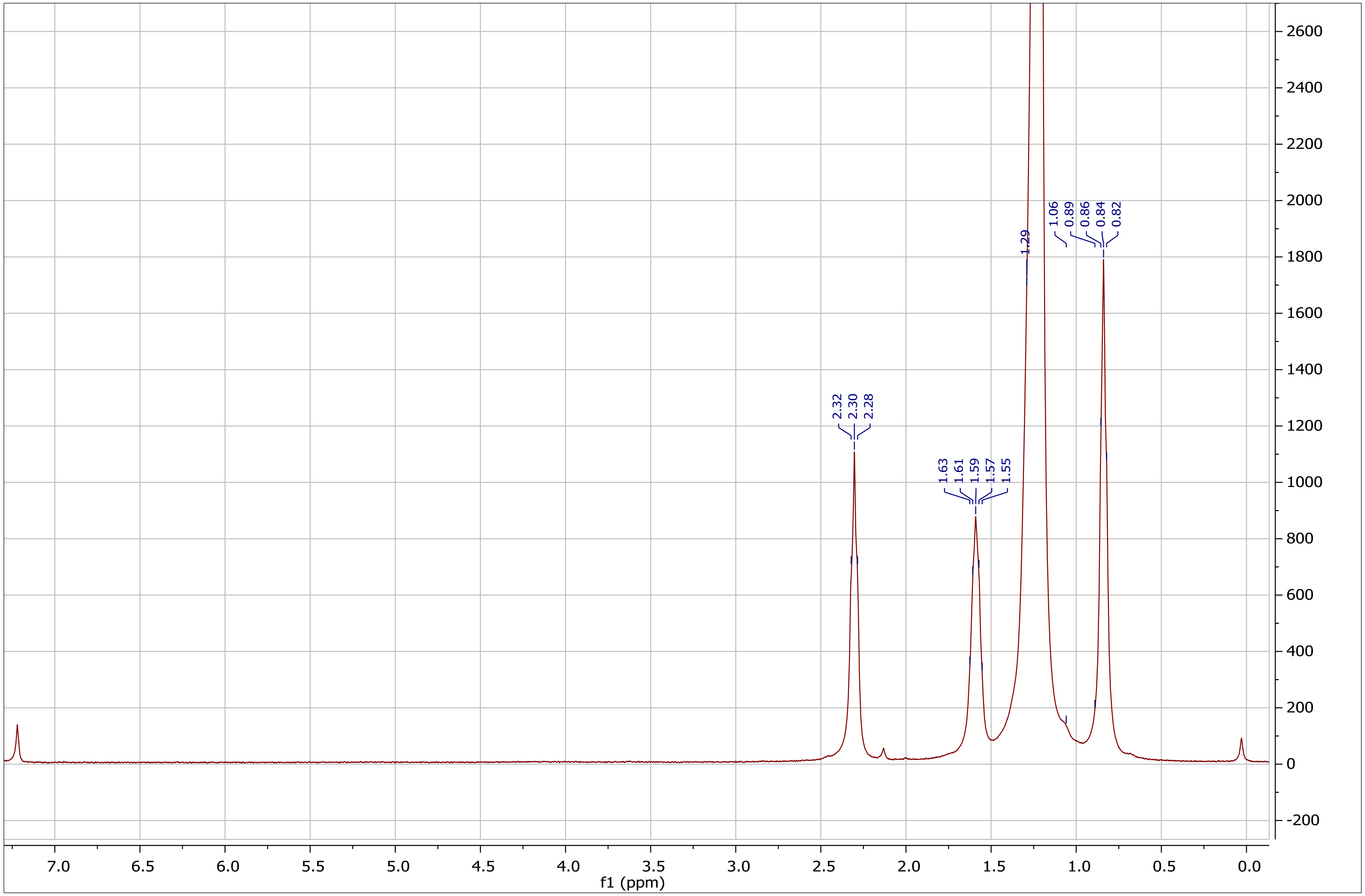

Figure S17. ^{1}H NMR spectra of compound **4**.

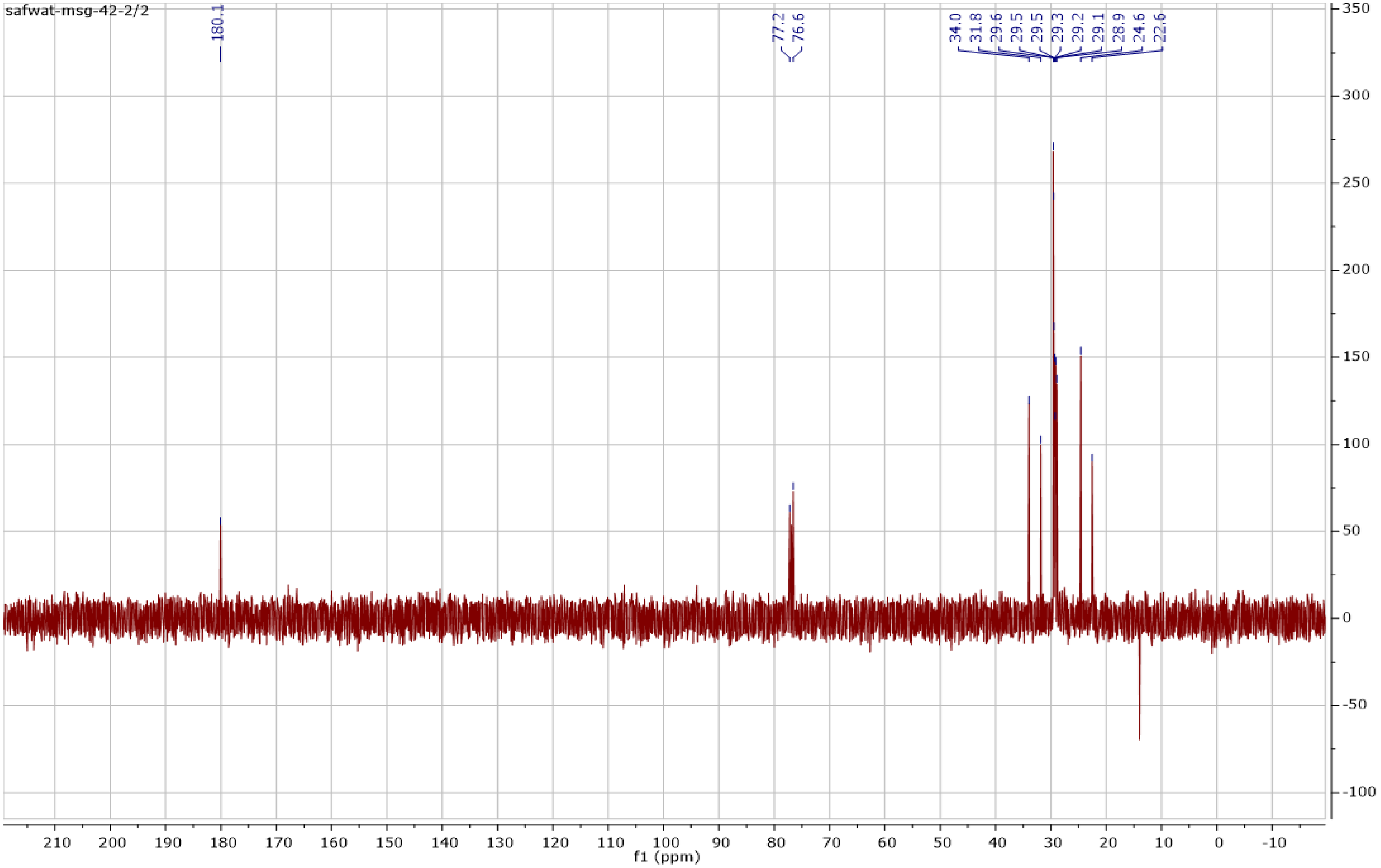

Figure S18. ^{13}C APT NMR spectra of compound **4**.

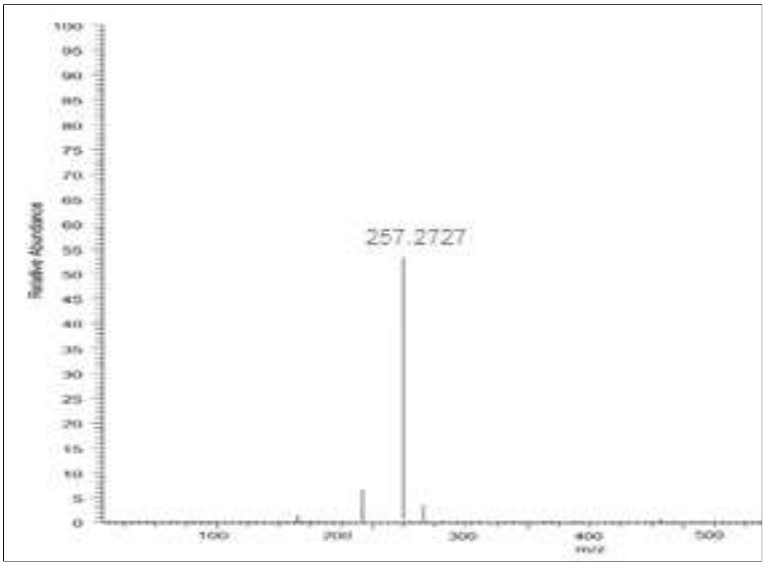

Figure S19. ESI-HRMS chromatogram of compound **5**.

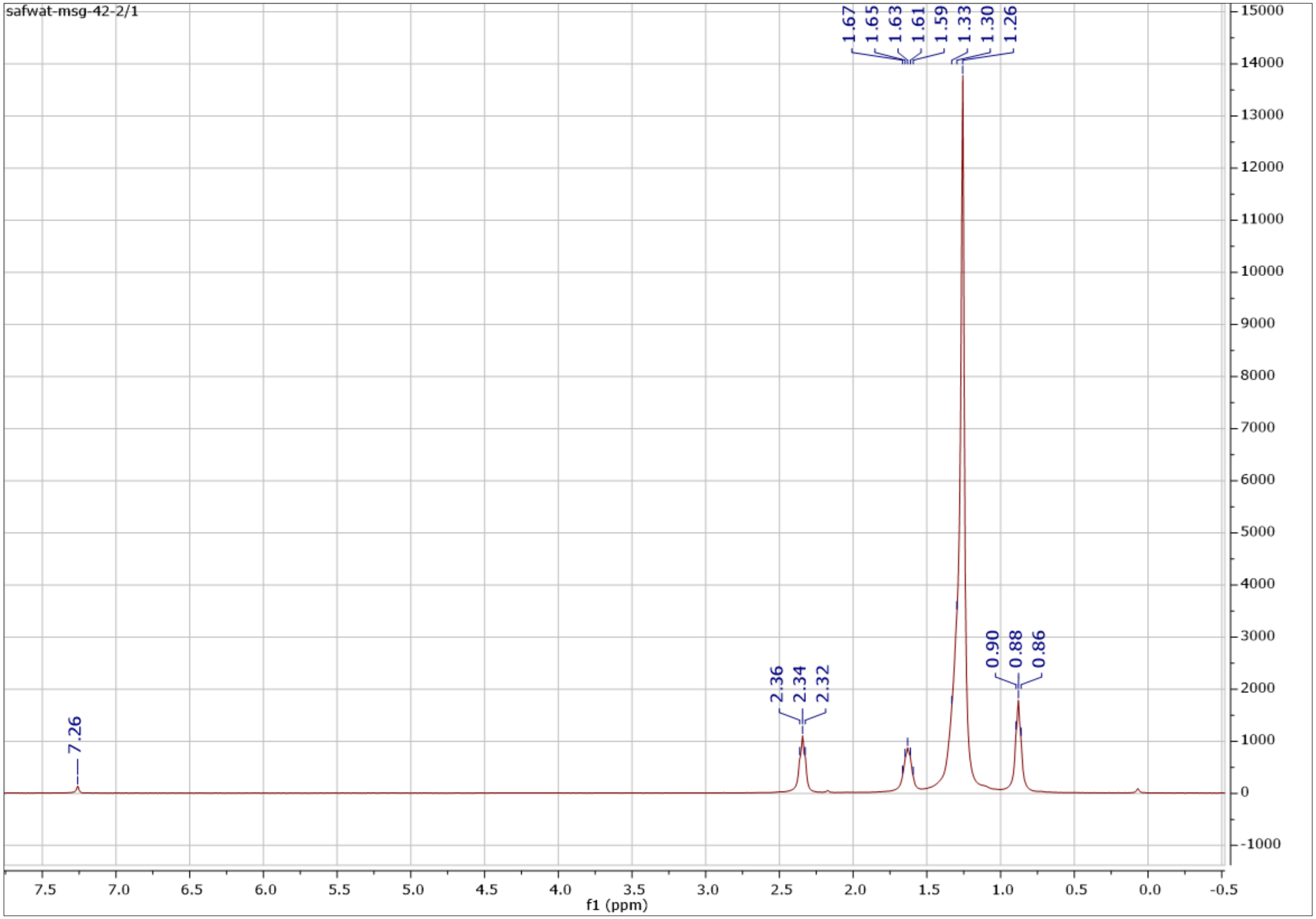

Figure S20. ^{1}H NMR spectra of compound **5**.

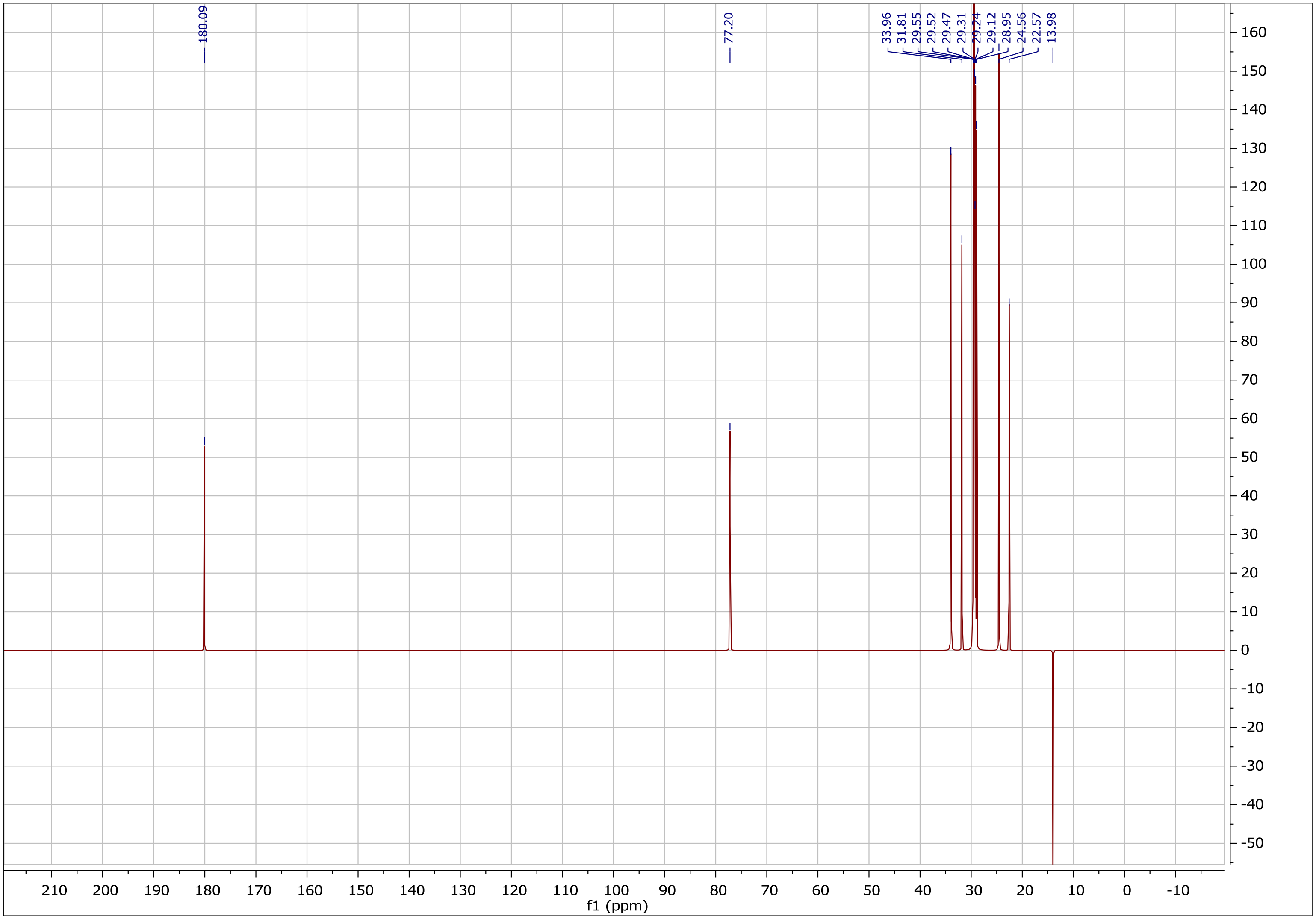

Figure S21. ^{13}C APT NMR spectra of compound **5**.

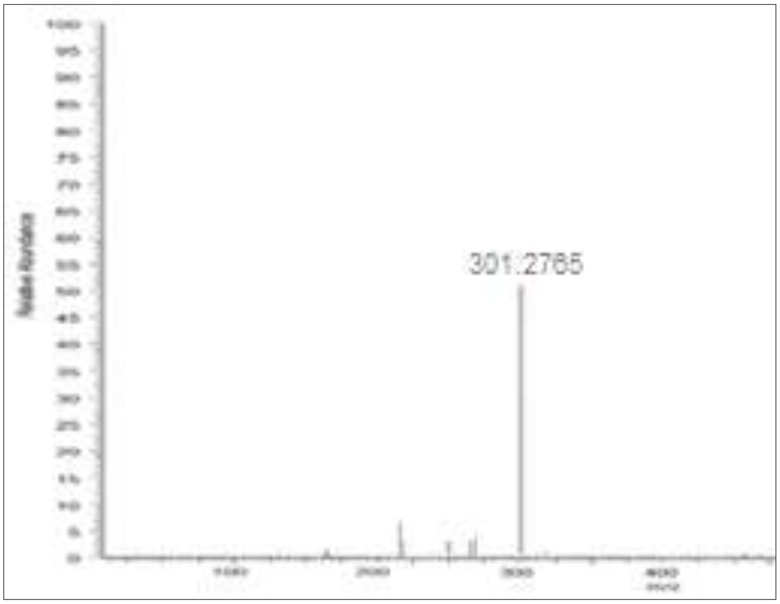

Figure S22. ESI-HRMS chromatogram of compound **6**.

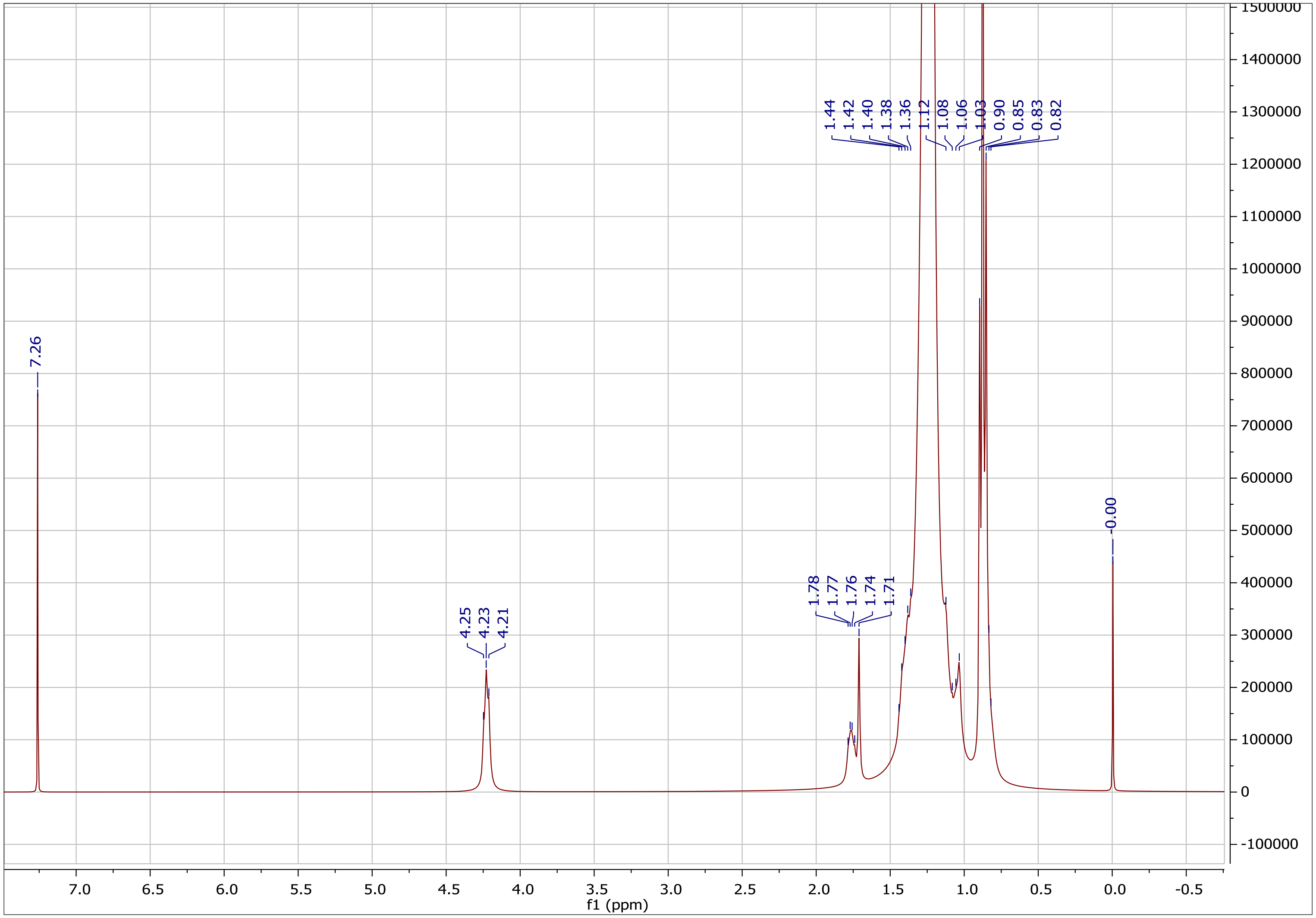

Figure S23. ^{1}H NMR spectra of compound **6**.

Table S1. Liver enzymes and kidney markers in the study groups.

	ALT (IU/L)	AST (IU/L)	Urea (mg/dL)	Creatinine (mg/dL)
Normal	44.60 ± 4.29	53.78 ± 6.31	18.52 ± 3.22	0.90 ± 0.15
Control	53.59 ± 6.11	63.61 ± 6.90	25.33 ± 5.60	1.30 ± 0.25
Ceramide 1 (1 mg/kg)	51.23 ± 4.10	62.31 ± 5.40	25.32 ± 6.66	1.25 ± 0.24
Ceramide 1 (2 mg/kg)	50.31 ± 5.91	60.40 ± 5.70	24.76 ± 6.87	1.22 ± 0.27
Ceramide 2 (1 mg/kg)	52.51 ± 5.10	61.33 ± 5.89	24.83 ± 6.50	1.24 ± 0.27
Ceramide 2 (2 mg/kg)	49.32 ± 6.90	60.32 ± 7.10	23.60 ± 6.98	1.21 ± 0.26

Values are expressed as mean ± SD. Data were analyzed using ANOVA followed by Bonferroni post hoc test.
